# The hemicellulose-degrading enzyme system of the thermophilic bacterium *Clostridium stercorarium*: comparative characterisation and addition of new hemicellulolytic glycoside hydrolases

**DOI:** 10.1186/s13068-018-1228-3

**Published:** 2018-08-23

**Authors:** Jannis Broeker, Matthias Mechelke, Melanie Baudrexl, Denise Mennerich, Daniel Hornburg, Matthias Mann, Wolfgang H. Schwarz, Wolfgang Liebl, Vladimir V. Zverlov

**Affiliations:** 1 Department of Microbiology, TUM School of Life Sciences Weihenstephan, Technical University of Munich, Emil-Ramann-Str. 4, 85354 Freising, Germany; 2 Present Address: School of Medicine, Stanford University, Stanford, CA 94305 USA; 3 Max Planck Institute of Biochemistry, Am Klopferspitz 18, 82152 Martinsried, Germany; 4 Institute of Molecular Genetics, Russian Academy of Science, Kurchatov Sq. 2, Moscow, 123182 Russia

**Keywords:** Biomass degradation, Enzyme characterisation, Proteome analysis, Arabinoxylan, Xylanase, Substrate specificity, Product specificity

## Abstract

**Background:**

The bioconversion of lignocellulosic biomass in various industrial processes, such as the production of biofuels, requires the degradation of hemicellulose. *Clostridium stercorarium* is a thermophilic bacterium, well known for its outstanding hemicellulose-degrading capability. Its genome comprises about 50 genes for partially still uncharacterised thermostable hemicellulolytic enzymes. These are promising candidates for industrial applications.

**Results:**

To reveal the hemicellulose-degrading potential of 50 glycoside hydrolases, they were recombinantly produced and characterised. 46 of them were identified in the secretome of *C. stercorarium* cultivated on cellobiose. Xylanases Xyn11A, Xyn10B, Xyn10C, and cellulase Cel9Z were among the most abundant proteins. The secretome of *C. stercorarium* was active on xylan, β-glucan, xyloglucan, galactan, and glucomannan. In addition, the recombinant enzymes hydrolysed arabinan, mannan, and galactomannan. 20 enzymes are newly described, degrading xylan, galactan, arabinan, mannan, and aryl-glycosides of β-d-xylose, β-d-glucose, β-d-galactose, α-l-arabinofuranose, α-l-rhamnose, β-d-glucuronic acid, and *N*-acetyl-β-d-glucosamine. The activities of three enzymes with non-classified glycoside hydrolase (GH) family modules were determined. Xylanase Xyn105F and β-d-xylosidase Bxl31D showed activities not described so far for their GH families. 11 of the 13 polysaccharide-degrading enzymes were most active at pH 5.0 to pH 6.5 and at temperatures of 57–76 °C. Investigation of the substrate and product specificity of arabinoxylan-degrading enzymes revealed that only the GH10 xylanases were able to degrade arabinoxylooligosaccharides. While Xyn10C was inhibited by α-(1,2)-arabinosylations, Xyn10D showed a degradation pattern different to Xyn10B and Xyn10C. Xyn11A released longer degradation products than Xyn10B. Both tested arabinose-releasing enzymes, Arf51B and Axh43A, were able to hydrolyse single- as well as double-arabinosylated xylooligosaccharides.

**Conclusions:**

The obtained results lead to a better understanding of the hemicellulose-degrading capacity of *C. stercorarium* and its involved enzyme systems. Despite similar average activities measured by depolymerisation tests, a closer look revealed distinctive differences in the activities and specificities within an enzyme class. This may lead to synergistic effects and influence the enzyme choice for biotechnological applications. The newly characterised glycoside hydrolases can now serve as components of an enzyme platform for industrial applications in order to reconstitute synthetic enzyme systems for complete and optimised degradation of defined polysaccharides and hemicellulose.

**Electronic supplementary material:**

The online version of this article (10.1186/s13068-018-1228-3) contains supplementary material, which is available to authorized users.

## Background

Hemicelluloses are heterogeneous polysaccharides in plant cell walls that are characterised by β-(1,4)-linked backbones with an equatorial configuration [1]. After cellulose, they are the second most common polysaccharides representing about 20–35% of lignocellulosic biomass [2, 3]. Hemicelluloses have a high structural and chemical diversity. Their backbones are usually branched or substituted with short oligosaccharides, monosaccharides, and organic acids. The major building blocks are pentoses (xylose, arabinose), hexoses (glucose, galactose, mannose), sugar acids (glucuronic acid), acetic acid, and phenolic acids (ferulic acid, *p*-coumaric acid) [2, 4]. According to the predominant monosaccharide in the main polymeric chain, hemicelluloses are classified into four types of polysaccharides: xylans, mannans, β-(1,3;1,4)-glucans, and xyloglucans. Further polysaccharides, such as galactans, arabinans, and arabinogalactans, are rather parts of pectin substances and do not share the equatorial β-(1,4)-linked backbone structure [1, 5]. Because they are often isolated with the hemicelluloses, they are included in this study. Among hemicelluloses, xylans are the most abundant polysaccharides and the major hemicelluloses in hardwood and grasses [3, 6].

Effective enzymatic degradation of hemicelluloses is of great interest for various applications in industrial processes, such as efficient conversion of lignocellulosic biomass to fuels and chemicals, bleaching of paper pulp, and improvement of food properties [2]. The majority of hemicellulolytic enzymes belong to the glycoside hydrolases (GH), which hydrolyse the glycosidic bond between two carbohydrates or a carbohydrate and a non-carbohydrate moiety. Based on amino acid sequence and concomitant structural similarities, the Carbohydrate-Active Enzymes database (CAZy) classifies glycoside hydrolases and further carbohydrate-active enzymes, such as polysaccharide lyases (PL) and carbohydrate esterases (CE), into functional families [7, 8].

Saprophytic bacteria degrade lignocellulosic biomass by secreting glycoside hydrolases active on cellulose and hemicellulose [9]. One of the first identified and best characterised cellulolytic bacteria is *Clostridium stercorarium* [10, 11]. The Gram-positive, strictly anaerobic and thermophilic bacterium is well known for being a specialist in the hydrolysis of hemicellulose. *C. stercorarium* degrades polysaccharides with a variety of hemicellulolytic enzymes, ferments a wide range of sugars, hexoses as well as pentoses, and produces acetate, ethanol, CO_2_, and H_2_ [10–14]. Recently it was suggested to assign *C. stercorarium* to the new genus *Ruminiclostridium* and to rename it *Ruminiclostridium stercorarium* [15].

A number of *C. stercorarium* enzymes involved in the degradation of polysaccharides have already been characterised [12, 16, 17]. *C. stercorarium* produces two cellulases that form a two-component cellulase system: Cel9Z (avicelase I, GH9) and Cel48Y (avicelase II, GH48). Cel9Z, which combines endoglucanase and exoglucanase activities, and the exoglucanase Cel48Y act synergistically to degrade crystalline cellulose [18–22]. Three endo-xylanases of *C. stercorarium* have been described: Xyn11A, Xyn10B, and Xyn10C (sometimes referred to as Xyn10B) [23–25]. The screening of a genomic library revealed seven genes related to arabinoxylan hydrolysis, encoding the three xylanases, two α-l-arabinofuranosidases (Arf43A, Arf51B), and two β-d-xylosidases (Bxl39A, Bxl3B) [26]. All seven enzymes and the β-glucosidase Bgl3Z hydrolyse arabinoxylan or degradation products thereof. Four glycoside hydrolases without established involvement in cellulose or xylan degradation have been described: two β-xylosidases (Xyl43A and Xyl43B), an α-galactosidase (Aga36A), and an α-glucuronidase (Agu67A) [27–30]. The α-l-rhamnosidase Ram78A hydrolyses naringin to prunin and rhamnose and was applied for the production of rhamnose from citrus peel naringin [16, 31]. In addition, an extracellular feruloyl esterase, a pectate lyase (Pel9A), a cellobiose phosphorylase (CepA), and a cellodextrin phosphorylase (CepB) have been characterised [32–34].

Although *C. stercorarium* is one of the model organisms for carbohydrate-degrading bacteria, only a few individual enzymes and the enzyme systems for hydrolysis of cellulose and xylan have so far been described in detail. In addition, the bacterium produces a number of putative hemicellulolytic enzymes with still unidentified activity awaiting further characterisation for potential applications [35].

In this study, we investigated the majority of glycoside hydrolases from the genome of *C. stercorarium* DSM 8532 to reach a deeper insight into the polysaccharide-degrading machinery of this bacterium. A total of 50 proteins potentially involved in hemicellulose hydrolysis were screened for activity on numerous substrates, including hemicellulosic polysaccharides and related oligosaccharides, as well as various aryl-glycosides. We characterised the most promising enzymes for biotechnological applications and present a set of new enzymes for the degradation of xylan, xyloglucan, mannan, galacto- and glucomannan, arabinan, and galactan. The presented enzymes may function as a technology platform for utilisation of these polysaccharides through modification, partial or complete degradation.

## Methods

### Preparation of secreted *C. stercorarium* proteins

Culture supernatants were prepared from *C. stercorarium* cells grown on filter paper or oat spelt xylan as carbon source (5 g/L) in 500 mL GS2 medium until substrate depletion. The main culture was inoculated with 25 mL of a 50-mL starter culture [36]. Extracellular protein was precipitated from clarified culture with ammonium sulfate as described by Koeck et al. and resuspended in 15 mL saturated (NH_4_)_2_SO_4_ solution [37]. For enzymatic assays, 2 mL suspension was centrifuged (16,000*g*, 20 min) and precipitated protein was dissolved in 200 µL 0.1 M MOPS reaction buffer (pH 6.5, 50 mM NaCl, 10 mM CaCl_2_). Protein concentration was determined by using a Bradford Protein Assay kit (Thermo Fisher Scientific).

### Cloning, expression, and purification of recombinant enzymes

Glycoside hydrolases (GH) of interest were identified using the classification of the Carbohydrate-Active Enzymes database (CAZy) [8, 38]. Genes encoding putative hemicellulolytic glycoside hydrolase families were amplified from genomic DNA of *C. stercorarium* DSM 8532 (NC_020134.1) and cloned into pET-24c(+) vectors (Merck) using the Gibson assembly method (primer list in Additional file 1: Table S1) [15, 39, 40]. Genes were cloned without predicted N-terminal signal peptides (SignalP 4.1 server, default cutoff: 0.3) and fused to a C-terminal His_6_-tag for protein purification by immobilised metal ion affinity chromatography (IMAC) [41–43]. Enzyme production was realised in *Escherichia coli* BL 21 Star™ using ZYP-5052 auto-induction medium [44]. Protein production and protein purification were accomplished as described by Mechelke et al. [45]. After purification of the recombinant proteins by IMAC, remaining *E. coli* proteins were removed by heat denaturation (55 °C, 15 min). During the purification, samples were taken to confirm the size and purity of the proteins by sodium dodecyl sulfate-polyacrylamide gel electrophoresis (SDS-PAGE) [46]. The ExPASy ProtParam tool was used to calculate the extinction coefficient and the molecular weight of the purified recombinant enzyme [47, 48].

### Proteome analysis of intra- and extracellular glycoside hydrolases

We employed a quantitative mass spectrometry-based proteomics strategy to identify secreted and intracellular proteins of *C. stercorarium. C. stercorarium* was grown in 50 mL GS2 medium with 0.5% w/v cellobiose until early logarithmic phase. Bacterial cells were harvested by centrifugation (5000*g*, 15 min, 4 °C). The cell pellet was resuspended in 5 mL 20 mM MES buffer (pH 6.5). Extracellular protein was precipitated overnight as described above. The precipitate was resuspended in 150 µL supernatant. Proteins were isolated and digested as described by Hornburg et al. [49]. Peptides were desalted on C18 StageTips as described previously and subjected to LC MS/MS analysis [50].

LC–MS/MS: The peptides were separated via a nano HPLC (Thermo Fisher Scientific) employing a C18 column. The HPLC was directly coupled to quadrupole-Orbitrap mass spectrometer via a nano electrospray ion source (Q Exactive™, Thermo Fisher Scientific). HPLC and Q Exactive™ were operated as described previously [49, 50].

We processed the MS raw data with MaxQuant (v. 1.3.8.2) [51]. MS/MS spectra were searched against the *C. stercorarium* database containing 2688 forward entries as well as a list of common contaminants included in MaxQuant. Enzyme specificity was set to trypsin allowing cleavage N-terminally to proline and up to two miscleavages. The minimum length for peptides to be considered for identification was set to seven amino acids. Carbamidomethylation was defined as a fixed modification, and acetylation (N-terminus) and methionine oxidation were set as variable modifications. As false discovery rate (FDR) a cutoff of 1% was applied at the peptide and protein level. The median of replicates for protein length normalised log2 protein intensities (termed iBAQ values in MaxQuant) were used to compare protein abundances.

### Enzymatic activity and optimal reaction conditions

Activity determination was performed with *para*-nitrophenyl (*p*NP) glycosides and polysaccharide substrates (Additional file 1: Table S2). To determine their activity, the recombinant enzymes were incubated with 4 mM each of the 12 *p*NP-glycosides. In a first qualitative examination, reactions were performed with 10 mg/L enzyme overnight (18 h). Determined activities were quantified with 0.05–10 mg/L enzyme and reaction times between 5 min and 2 h. All reactions were carried out at 60 °C and pH 6.0 in a total volume of 50 µL or 500 µL using standard reaction buffer (100 mM MOPS, pH 6.5 at room temperature, 50 mM NaCl, 10 mM CaCl_2_). The reaction was stopped by addition of two volumes of 1 M Na_2_CO_3_ and absorption of *p*NP was measured at 405 nm. Activity was calculated by comparison to *p*NP as standard.

The hemicellulolytic activity of secreted or recombinantly produced enzymes was determined by measuring the increase of reducing groups caused by polysaccharide hydrolysis with the 3,5-dinitrosalicylic acid (DNSA) assay [52]. One unit (U) of enzymatic activity was defined as the amount of enzyme releasing 1 µmol of xylose equivalent per minute and was calculated using xylose as standard. Reactions were performed in a total volume of 150 µL in 96-well PCR plates with 0.5% w/v polysaccharide substrate, except for glucomannan (0.125%) and Avicel (0.25%). After incubation, the reaction mixture was centrifuged (1100*g*, 5 min, 4 °C) and reducing sugars were determined in a 50-µL sample. For qualitative activity determination, 5 mg/L of secreted protein or recombinant enzyme was incubated with the polysaccharides in standard reaction buffer at pH 6.5 and 60 °C for 16 h. Quantitative determination of the enzyme activity was realised using selected polysaccharides with 0.05–24 mg/L of recombinant enzyme and reaction times between 30 min and 2 h. The pH dependence of enzyme activity was determined in 100 mM citrate-phosphate buffer (citric acid, Na_2_HPO_4_, 50 mM NaCl) with pH between pH 4.0 and pH 8.0 (pH at 60 °C) in steps of 0.5 at 60 °C. Temperature dependence of enzyme activity was also determined in 100 mM citrate-phosphate buffer at each enzyme’s optimal pH. 10 different points were measured over a temperature gradient spanning 30 °C. The gradient was chosen depending on the temperature optimum of the enzyme. For the determination of the specific activity of the enzymes, hydrolysis was performed in 100 mM MOPS buffer (50 mM NaCl, 10 mM CaCl_2_) at pH 6.0 or 6.5 (Abn43A) and optimal temperature. All enzymatic assays were performed in triplicate using a xylose calibration curve.

### Substrate specificity on oligosaccharides

Selected enzymes involved in xylan degradation were incubated with xylopentaose (X_5_) and a number of arabinoxylan oligosaccharides (AXOS): 3^2^-α-l-arabinofuranosyl-xylobiose (A^3^X), 2^3^-α-l-arabinofuranosyl-xylotriose (A^2^XX), 2^3^,3^3^-di-α-l-arabinofuranosyl-xylotriose (A^2+3^XX), 2^3^,3^3^-di-α-l-arabinofuranosyl-xylotetraose (XA^2+3^XX), 3^3^-α-l-arabinofuranosyl-xylotetraose (XA^3^XX), and a mixture of XA^3^XX and 2^3^-α-l-arabinofuranosyl-xylotetraose (XA^2^XX) (Megazyme). Hydrolysis was performed overnight at optimal pH and temperature of each enzyme, with 2 mg/L enzyme and 20 mg/L oligosaccharide or oligosaccharide mixture in 500 µL volume. The degradation products were analysed using high-performance anion-exchange chromatography with pulsed amperometric detection (HPAEC-PAD) as described by Mechelke et al. [45].

### Chromatographic analysis of poly- and oligosaccharide hydrolysates

Analysis of poly- and oligosaccharide hydrolysis products was carried out with thin-layer chromatography (TLC) or high-performance anion-exchange chromatography with pulsed amperometric detection (HPAEC-PAD). Hydrolysates were centrifuged (16,000*g*, 10 min) prior to analysis. The volume for 1 µg xylose equivalent, determined by DNSA assay, was separated by TLC as described by Zverlov et al. [53]. Analysis with HPAEC-PAD was performed with an ICS 3000 Dionex system (Thermo Fisher Scientific) equipped with a CarboPac™ PA1 column and a linear sodium acetate (NaOAc) gradient as described by Mechelke et al. [45]. Hydrolysates were diluted tenfold with double-distilled water and supplemented with 2 mg/L d-mannitol as internal standard (ISTD). Degradation products were identified by comparing them to a number of monosaccharide (2 mg/L) and oligosaccharide (10 mg/L) standards: arabinose (A), xylose (X), xylobiose (X_2_), -triose (X_3_), -tetraose (X_4_), -pentaose (X_5_), and arabinoxylooligosaccharides (AXOS) as listed above.

## Results

### Hemicellulose-degrading potential of the extracellular enzymes of *C. stercorarium*

**Fig. 1:**
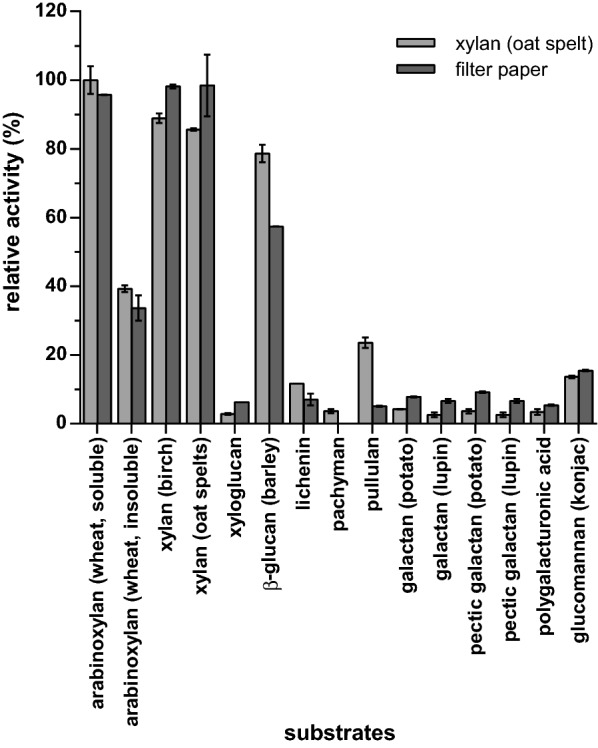
Relative activity of the secreted enzymes of *C. stercorarium* grown on oat spelt xylan (light grey) or filter paper (dark grey). Enzymatic activity was determined by DNSA-based assay. 5.0 mg/L extracellular enzymes, precipitated from the culture supernatant, were incubated with 0.5% (w/v) polysaccharide at pH 6.5 and 60 °C in 0.1 M MOPS reaction buffer overnight (16 h). Hydrolytic activity from oat spelt xylan medium on soluble wheat arabinoxylan was set to 100%. Tested polysaccharides with no activity were: arabinan, arabinogalactan (larch), curdlan, galactomannan (guar), gum arabic (acacia), inulin (dhalia tubers), laminarin, mannan, mannan (ivory nut), rhamnogalacturonan I, sinistrin

The hemicellulose-degrading capabilities of *C. stercorarium* derive from the glycoside hydrolases secreted by the bacterium into the extracellular environment (secretome). To determine their activity, secreted proteins were prepared from *C. stercorarium* grown on oat spelt xylan or filter paper. Both protein preparations displayed activities on 15 of 26 screened polysaccharides (Fig. 1). The highest activity, not only for the xylan preparation but also for the filter paper preparation, was found with xylan substrates such as wheat arabinoxylan (medium viscosity), birch wood xylan, and oat spelt xylan.

The activity on insoluble wheat arabinoxylan was 60% lower than on soluble wheat arabinoxylan (set to 100%). Compared with their xylanolytic activity the secreted enzymes had merely low activity on glucomannan, various galactans, xyloglucan, and polygalacturonic acid. Interestingly, the xylan protein preparation displayed higher relative activity on mixed linkage glucans, especially on barley β-glucan (79%) and pullulan (24%), compared to the enzyme preparation from filter paper medium (57% and 5%). While the determined activity of the xylan protein preparation was higher for mixed linkage glucans, the protein preparation from filter paper appeared to be more active on birch and oat spelt xylan. *C. stercorarium* grew significantly faster and to a higher optical density in medium with oat spelt xylan and produced a 3.1-fold higher amount of precipitated protein (3.62 mg/L vs. 1.17 mg/L). The polysaccharide substrates for the activity screening of the single recombinant enzymes were selected based on the determined activities of the extracellular enzymes.

### Selection of hemicellulolytic enzymes

The Carbohydrate-Active Enzymes database (CAZy) lists 71 proteins with glycoside hydrolase (GH) modules for *C. stercorarium*, distributed over 34 GH families [8, 38]. Based on their predicted activities, 24 typical hemicellulolytic GH families were selected and 50 genes containing modules of these GH families were heterologously expressed in *E. coli* for further investigation. Nineteen enzymes of 10 GH families were excluded, because enzymes of these GH families require NAD + (GH4), are mostly parts of starch (GH13, GH15), chitin (GH18), peptidoglycan (GH23), and cellulose degradation (GH48), or act as phosphorylases. Two enzymes of GH family 36, usually α-galactosidases, were also excluded (Additional file 1: Table S3).

All 50 proteins were successfully cloned, produced and purified in intact form (Table 1, Additional file 1: Figure S1). The amount of purified protein ranged from 1.8 mg/L *E. coli* culture for BglA to 253.0 mg/L for Bxl39A. One protein, the endo-β-1,4-galactanase Gal53A was produced in a shortened version with only 909 amino acids (AA) instead of the complete 1784 AA, because the full-length gene could not be cloned into the pET-24c(+) expression vector. PCR oligonucleotide primer was designed to bind 1296 bp behind the 5′ end and 1329 bp in front of the 3′ end of the *galA* gene to include the complete GH53 module while excluding the S-layer homology modules (Additional file 1: Figure S2). Signal peptides, with lengths between 13 AA and 34 AA, were predicted for 11 proteins, of which 10 were present in the secretome of *C. stercorarium* [43]. The enzyme Axh43A was detected neither in the secretome nor in the intracellular proteome.

**Table 1:** List of investigated glycoside hydrolases of *C. stercorarium*

Accession number	Protein name	Modules	SP	Lit.
AGC67981.1	Bga2B	GH2	–	
AGC67186.1	Bga2E	GH2	–	
AGC67268.1	Bga2D	GH2	–	
AGC67637.1	Bga2C	GH2	–	
AGC68382.1	Uid2A	GH2	–	
AGC67275.1	Bxl3B	GH3	–	[17]
AGC67337.1	Bgl3Z	GH3	–	[17]
AGC67350.1	–	GH3	–	
AGC68204.1	–	GH3, CBM6	–	
AGC68338.1	Nag3A	GH3	22 AA	
AGC68873.1	Cel9Z	GH9, 2× CBM3, 2× CBMX2	27 AA	[19, 20]
AGC67515.1	Xyn10E	GH10	–	
AGC67677.1	Xyn10C	GH10, CBM9, 2× CBM22	29 AA	[17, 25]
AGC67715.1	Xyn10B	GH10	34 AA	[17, 24]
AGC67759.1	Xyn10D	GH10	–	
AGC68909.1	Xyn11A	GH11, 3× CBM6	30 AA	[17, 23]
AGC68130.1	Man26A	GH26, CBM35	26 AA	
AGC68671.1	–	GH27	–	
AGC67830.1	–	GH28	–	
AGC67947.1	–	GH28	–	
AGC67128.1	–	GH29	–	
AGC68208.1	Bxl31D	GH31	–	
AGC69232.1	Bga35A	GH35	–	
AGC68033.1	–	GH38	–	
AGC67890.1	Bxl39A	GH39	–	[17]
AGC67716.1	Axh43A	2× GH43, CBM6	29 AA	
AGC67945.1	Arf43A	GH43	–	[17]
AGC68110.1	Arf43C	GH43	–	
AGC68111.1	Abn43A	GH43	31 AA	
AGC67521.1	Xyl43B	GH43	–	[28]
AGC67885.1	Xyl43A	GH43	–	[27]
AGC69509.1	Bxl43C	GH43	–	
AGC67626.1	Arf51B	GH51	–	[17, 56]
AGC68692.1	Gal53A	GH53, 4× CBM61	33 AA	
AGC69355.1	Agu67A	GH67	–	[30]
AGC68061.1	Ram78A	GH78	13 AA	[16, 31]
AGC69452.1	–	GH88	–	
AGC67127.1	–	GH95	–	
AGC68039.1	Xyn105F	GH105	–	
AGC67892.1	–	GH105	–	
AGC67946.1	–	GH105	–	
AGC68044.1	–	GH105	–	
AGC68046.1	–	GH105	–	
AGC67967.1	–	GH115	25 AA	
AGC67053.1	–	GH127	–	
AGC67292.1	–	GH127	–	
AGC67072.1	RamB	GHnc	–	
AGC68062.1	BglA	GHnc	–	
AGC69032.1	–	GHnc	–	
AGC69275.1	ArfD	GHnc	–	

GenBank accession number, GH family, CBMs, and predicted signal peptide (SP) are listed; enzymes with proven activity are additionally listed with protein name. CBM modules were obtained from the CAZy and the Pfam database [8, 57, 58]. Literature (Lit.) to previously characterised enzymes is indicated. Signal peptides were predicted with the SignalP 4.1 server and a default cutoff value of 0.3 [42]. *GHnc* Non-classified glycoside hydrolases

Thirty-two of the 50 investigated proteins showed hydrolytic activity on polysaccharides or *p*NP-glycosides among the substrates tested. No activity could be found for proteins containing modules of GH family 27, 28, 29, 38, 67, 88, 95, 115, or 127, two proteins containing a GH3 module, four proteins containing a GH105 module, and one protein with a non-classified GH module. Proteins which were characterised for the first time were named according to their main activity described herein, as was suggested by Schwarz et al. [54]. Schematic module structures of the examined proteins are given in Additional file 1: Figure S1. Seven proteins contained additional carbohydrate-binding modules (CBM) that are predicted to bind to polysaccharides, promoting the enzymatic degradation of insoluble polysaccharides [55].

### Glycoside hydrolases in the secretome and intracellular proteome of *C. stercorarium*

To examine if the heterologously produced enzymes of this study are also produced by *C. stercorarium*, the secretome and intracellular proteome of *C. stercorarium* were analysed by mass spectrometry (Additional file 1: Table S4, Additional file 2). The proteome analysis revealed 46 proteins produced by *C. stercorarium*, showing that these proteins may have a biological relevance in the organism. *C. stercorarium* did not produce the four enzymes Axh43A, Xyn105F, BglA, and AGC67892.1, at least when cultivated on cellobiose as carbon source. The two xylanases Xyn11A and Xyn10B were completely secreted while the four proteins, Bxl31D, Arf43C, AGC67127.1, and AGC68046.1, were only present in the intracellular proteome. The endo-acting enzymes Cel9Z, Xyn11A, Xyn10C, and Xyn10B ranked among the most abundant proteins (35%) in the secretome, while they were not detected in the intracellular proteome (Xyn11A, Xyn10B) or belonged to the 5% (Cel9Z) and 40% (Xyn10C) least abundant proteins of the intracellular proteome. These four enzymes, as well as Gal53A and Man26A, two enzymes ranking middle in the secretome and among the 10% of most rare proteins in the intracellular proteome, are predicted to be produced with N-terminal signal peptides and, therefore, can be regarded as secretory proteins. This indicates that *C. stercorarium* has an effective secretion system for endo-acting glycoside hydrolases, especially for the most important xylanases. Although 32 of the studied proteins do not appear to have a signal peptide, they were found in the secretome. This might be due to the release of proteins from cells by cell lysis or to alternative secretion pathways.

### Activity of hemicellulolytic enzymes on *p*NP-glycosides and polysaccharides

The activity screenings revealed 20 new *C. stercorarium* glycoside hydrolases which are here characterised the first time, including three enzymes with non-classified, possibly new GH modules. The recombinant enzymes exhibited activities for the same polysaccharides as the secretome, except for pachyman, pullulan, and polygalacturonic acid, and in addition, the enzymes Abn43A and Man26A hydrolysed arabinan and mannans, respectively. One enzyme with a GH105 module, Xyn105F, displayed xylanase activity, which is the first description of this activity for GH family 105.

In activity screenings with *p*NP-glycosides, 27 of the 32 enzymes with determined activity were able to hydrolyse the glycosidic bond in one or more of the artificial substrates. The assay revealed three α-l-arabinofuranosidases (Arf51B, Arf43C, ArfD), two β-d-glucosidases (BglA, Bgl3Z), three β-d-galactosidases (Bga2C, Bga2D, Bga2E), two α-l-rhamnosidases (Ram78A, RamB), seven β-d-xylosidases (Arf43A, Bxl39A, Bxl3B, Bxl43C, Bxl31D, Xyl43A, Xyl43B), one β-d-glucuronidase (Uid2A), and one *N*-acetyl-β-d-glucosaminidase (Nag3A), which showed their main activities towards *p*NP-glycosides (Table 2). Arf51B showed by far the highest activity of all studied enzymes, 208 U/mg for *p*NP-α-l-arabinofuranoside, followed by the activity of Bgl3Z for *p*NP-β-d-glucopyranoside (19.7 U/mg). More than half of the enzymes had side activities for further *p*NP-glycosides. The xylanases Xyn10D, Xyn10E, and Xyn105F, the arabinanase Abn43A, and the enzymes RamB and Xyl43A showed insignificant activities under the employed standard assay conditions. In a further assay the cell extract of *E. coli* producing RamB was incubated overnight with *p*NP-α-l-rhamnopyranoside at three different temperatures. Whereas the activity of the RamB preparation proved to be low at 60 °C (0.04 U/mg), it was 2.3-fold (0.10 U/mg) and 9.7-fold higher (0.43 U/mg) at 55 °C and 50 °C, respectively.

**Table 2:** Enzyme activity of examined glycoside hydrolyses on *para*-nitrophenyl linked glycosides (*p*NP-substrates)

Enzyme	Specific enzyme activity (U/mg)							
	*para*-Nitrophenyl-							
	α-l-Arabinofuranoside	α-d-Glucopyranoside	β-d-Glucopyranoside	β-d-Galactopyranoside	α-l-Rhamnopyranoside	β-d-Xylopyranoside	β-d-Glucuronide	*N*-acetyl-β-d-glucosaminide
Bga2B	–	–	–	++	–	–	–	–
Bga2E	–	–	–	+	–	–	–	–
Bga2D	–	–	–	+	–	–	–	nt
Bga2C	–	–	–	++	–	–	–	nt
Uid2A	(+)	–	+	+	–	(+)	+++	nt
Bxl3B	++	–	+	–	–	+++	–	–
Bgl3Z	+	(+)	+++	+	–	+++	–	–
Nag3A	–	–	–	–	–	–	+*	+
Xyn10E	–	–	–	(+)	–	(+)	–	nt
Xyn10C	(+)	–	(+)	–	–	+	–	nt
Xyn10B	(+)	–	(+)	(+)	–	++	–	nt
Xyn10D	–	–	–	–	–	(+)	–	nt
Bxl31D	–	–	–	–	–	+	–	nt
Bxl39A	+	–	–	–	–	+	–	nt
Axh43A	++	–	–	–	–	–	–	nt
Arf43A	(+)	–	(+)	–	–	++	–	nt
Arf43C	++	–	–	–	–	(+)	–	nt
Abn43A	(+)	–	–	–	–	–	–	nt
Xyl43B	–	–	–	–	–	+	–	nt
Xyl43A	(+)	–	–	–	–	(+)	–	nt
Bxl43C	+	–	–	–	–	++	–	nt
Arf51B	++++	–	–	–	–	+	–	nt
Ram78A	(+)	–	+	–	+++	–	–	nt
Xyn105F	–	–	–	(+)	–	–	–	nt
RamB	–	–	–	–	(+)	–	–	nt
BglA	–	–	+	–	–	(+)	–	nt
ArfD	+	–	(+)	+	–	–	–	nt

Hydrolysis experiments were performed in triplicates at pH 6.5 and 60 °C in 0.1 M MOPS reaction puffer for incubation times between 10 min and up to 2 h. Enzyme activity was determined by spectrophotometric detection of released *p*NP. No activity was detected for 22 of 49 enzymes (not listed). Gal53A was not tested. Tested aryl-glucosides with no activity were: pNP-α-d-galactopyranoside, pNP-α-d-mannopyranoside, pNP-β-d-mannopyranoside, and pNP-α-d-xylopyranoside. Substrate was not tested (nt), incubation overnight (*), detectable but very low activity ((+)), 0.05–0.5 U/mg (+), 0.5–5.0 U/mg (++), > 5.0 U/mg (+++), > 100.0 U/mg (++++)

**Fig. 2:**
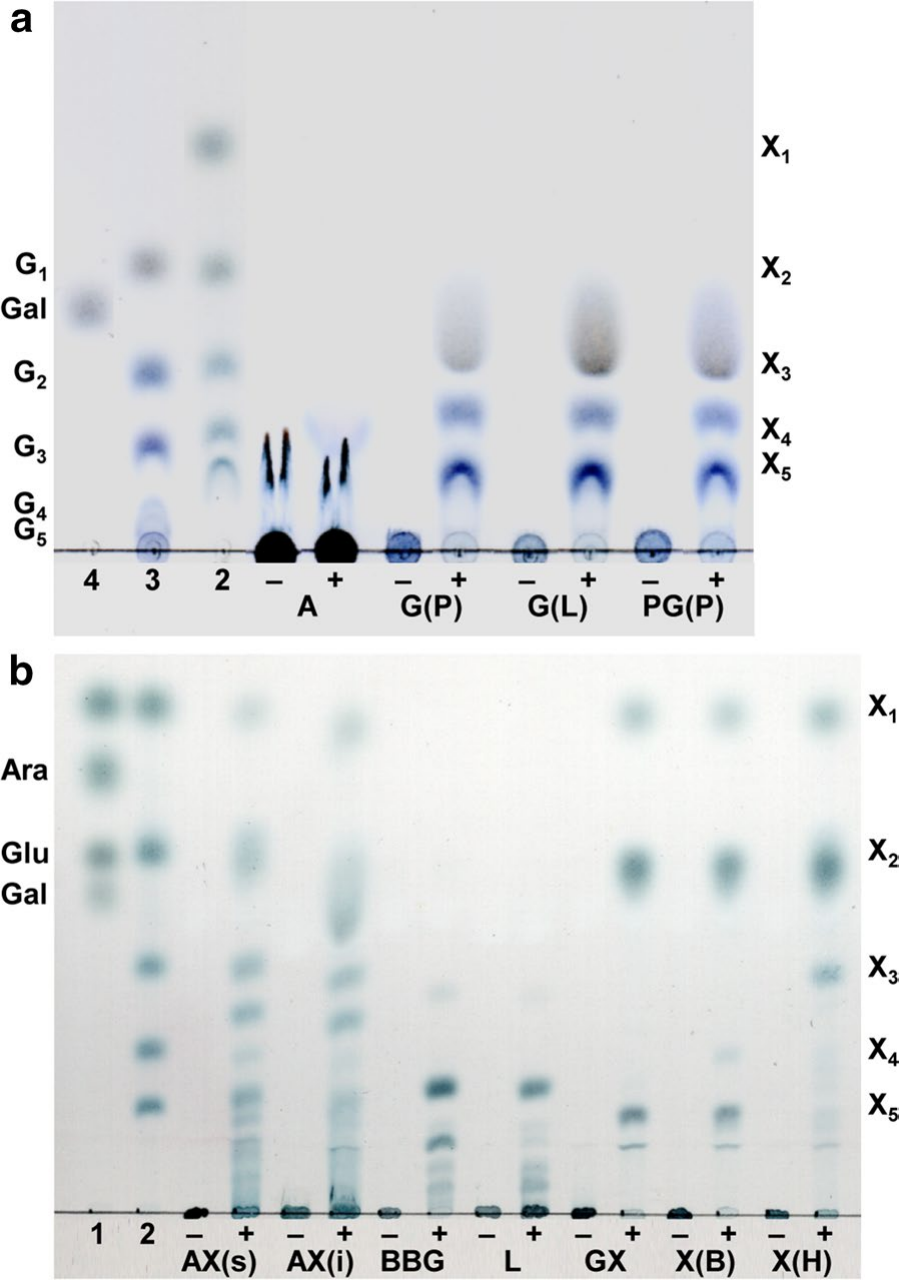
Hydrolytic products of different polysaccharides analysed by thin-layer chromatography (TLC). Polysaccharides were hydrolysed by **a** Gal53A or **b** Xyn10B. Samples were incubated overnight (16 h) at pH 6.5 and 60 °C with 5.0 mg/L enzyme and 0.5% substrate in 0.1 M MOPS reaction buffer. Arabinan (A), galactan potato (G(P)), galactan lupin (G(L)), pectic galactan potato (PG(P)), soluble wheat arabinoxylan (AX(s)), insoluble wheat arabinoxylan (AX(i)), barley β-glucan (BBG), lichenin (L), glucuronoxylan (GX), xylan (birch) (X(B)), xylan (oat spelt) (X(H)), negative control (−), hydrolysate (+), standards: xylose, arabinose, glucose, galactose (1), xylose, xylobiose, xylotriose, xylotetraose, xylopentatose (2), glucose, cellobiose, cellotriose, cellotetraose, cellopentose (3), galactose (4)

An overnight screening with 29 different polysaccharides at 60 °C showed that 17 of the studied enzymes catalyse the release of reducing sugars from 16 of the polysaccharides. The enzymes containing modules of the GH families 2, 3, 9, 10, 11, 26, 35, 43, 51, 53, or 105 hydrolysed either one or more of the xylan, glucan, galactan, and mannan-based polysaccharides, and xyloglucan. Further analysis of the hydrolytic products by TLC identified the enzymes as two β-d-galactosidases (Bga35A, Bga2B), two β-d-xylosidases (Bxl3B, Arf43A), one endo-glucanase (Cel9Z), six xylanases (Xyn11A, Xyn10B, Xyn10C, Xyn10D, Xyn10E, Xyn105F), one mannanase (Man26A), one arabinoxylan arabinofuranohydrolase (Axh43A), two α-l-arabinofuranosidases (Arf51B, Arf43C), one arabinanase (Abn43A), and one endo-galactanase (Gal53A) (Fig. 2, Additional file 1: Figure S3).

The specific activities of the glycoside hydrolases were determined at the temperature optimum (under standard assay conditions) of each enzyme (Table 3). The galactanase Gal53A degrading the β-(1,4)-galactan backbone of potato galactan, lupin galactan, and potato pectic galactan released short-chain oligosaccharides, while the β-d-galactosidases were exo-acting enzymes and released galactose as the only product (Additional file 1: Figure S3). Bga35A cleaved galactose off potato and lupin galactan and pectic galactan. Bga2B, on the other hand, released galactose only from potato galactan but also cleaved off the terminal β-(1,2)-linked galactose from galactosyl-xylose side chains that are attached with α-(1,6)-linkages to the backbone glucose residues of xyloglucan.

**Table 3:** Hemicellulolytic activity of seventeen glycoside hydrolases on 29 different polysaccharides

Enzymes	Specific activity (U/mg)							
	Arabinan	Arabinoxylan (wheat, soluble)	Arabinoxylan (wheat, insoluble)	4-*O*-Methyl-glucuron-oxylan	Xylan (birch)	Xylan (oat spelt)	Xyloglucan	β-Glucan (barley)
Bga2B	(+)	–	–	–	–	–	3.11 ± 0.04	–
Bxl3B	–	0.25 ± 0.01	+	0.21 ± 0.01	0.28 ± 0.01	0.35 ± 0.02	–	–
Cel9Z	–	–	–	(+)	–	(+)	–	159 ± 1
Xyn10E	–	0.17 ± 0.01	–	+	(+)	(+)	–	–
Xyn10C	–	134 ± 5	14.1 ± 0.8	91.3 ± 3.8	56.3 ± 2.0	86.6 ± 1.2	–	19.1 ± 0.1
Xyn10B	–	597 ± 46	40.4 ± 3.7	483 ± 45	185 ± 20	679 ± 41	–	18.8 ± 1.6
Xyn10D	–	6.55 ± 0.09	–	+	1.24 ± 0.04	6.17 ± 0.08	–	(+)
Xyn11A	–	1030 ± 50	226 ± 47	936 ± 19	619 ± 7	937 ± 6	–	–
Man26A	–	–	–	–	–	–	+	–
Bga35A	(+)	–	–	–	–	–	–	–
Axh43A	+	41.5 ± 0.8	(+)	(+)	–	+	–	–
Arf43A	–	(+)	–	(+)	(+)	(+)	–	–
Arf43C	0.82 ± 0.06	–	–	–	–	–	–	–
Abn43A	79.5 ± 0.9	–	–	–	–	–	–	–
Arf51B	2.01 ± 0.03	0.32 ± 0.01	–	–	–	(+)	–	–
Gal53A	+	–	–	–	–	–	–	–
Xyn105F	–	0.22 ± 0.01	–	+	+	+	–	–

Enzymes	Specific activity (U/mg)							
	Lichenin	Galactan (potato)	Galactan (lupin)	Pectic galactan (potato)	Pectic galactan (lupin)	Mannan	Galactomannan (guar)	Glucomannan (konjac)
Bga2B	–	+	(+)	(+)	(+)	–	–	–
Bxl3B	–	–	–	–	–	–	–	–
Cel9Z	136 ± 9	–	–	–	–	–	–	32.9 ± 1.0
Xyn10E	–	–	–	–	–	–	–	–
Xyn10C	7.85 ± 0.45	–	–	–	(+)	–	–	+
Xyn10B	9.65 ± 0.65	–	–	–	(+)	–	–	(+)
Xyn10D	–	–	–	–	(+)	–	–	–
Xyn11A	–	–	–	–	–	–	–	–
Man26A	–	–	–	–	–	39.9 ± 2.0	216 ± 5	144 ± 3
Bga35A	–	2.17 ± 0.03	1.24 ± 0.08	1.08 ± 0.01	1.14 ± 0.02	–	–	–
Axh43A	–	–	–	–	(+)	–	–	–
Arf43A	–	–	–	–	(+)	–	–	–
Arf43C	–	–	1.99 ± 0.20	6.50 ± 0.13	4.46 ± 0.09	–	–	–
Abn43A	–	–	10.1 ± 0.3	21.3 ± 0.2	12.0 ± 2.1	–	–	–
Arf51B	–	–	–	(+)	–	–	–	–
Gal53A	–	+	+	+	+	+	–	–
Xyn105F	–	–	–	–	–	–	–	–

Hydrolysis experiments were performed in 0.1 M MOPS reaction buffer with 0.05–24 mg/L enzyme at pH 6.0 (pH 6.5 for Abn43A) and the temperature optimum of each enzyme for incubation times from 30 min up to 2 h. The specific enzyme activity was determined using DNSA assay in triplicates. Further tested polysaccharides for which no activity could be determined were: arabinogalactan (larch), curdlan, gum arabic (acacia), inulin (dahlia tubers), laminarin, mannan (ivory nut), pachyman, polygalacturonic acid, pullulan, rhamnogalacturonan I, sinistrin, chitosan and Avicel. No activity (–), detectable but very low activity ((+)), activity overnight (+), specific activity (numbers)

All six xylanases acted as endo-xylanases and showed their highest specific activity for soluble wheat arabinoxylan. Xyn10B and Xyn10C, both containing a GH10 module, additionally hydrolysed the β-(1,3;1,4)-glucan backbone of barley β-glucan and lichenin. While the activity of Xyn10B for arabinoxylan was 4.6 times higher than that of Xyn10C, the activities for β-glucan and lichenin were nearly the same. The xylooligosaccharides released by the xylanases differ in length: Xyn10B and Xyn10C mainly produced xylose and xylobiose while Xyn11A, containing a GH11 module, mainly produced xylobiose and xylotriose. All three enzymes released a number of additional oligosaccharides during the hydrolysis of arabinoxylan (Additional file 1: Figure S3). Xyn11A showed the highest specific activity (1030 U/mg), which was 1.7 times and 7.7 times higher than the specific activities of Xyn10B (597 U/mg) and Xyn10C (134 U/mg), respectively. The xylanases Xyn10D, Xyn10E, and Xyn105F also degraded xylan, but no activity was determined with β-glucan.

Cel9Z hydrolysed barley β-glucan and lichenin to oligosaccharides and degraded the backbone of glucomannan, which is composed of β-(1,4)-linked d-mannose and d-glucose. The arabinanase Abn43A cleaved the α-(1,5)-linked backbone of arabinan and released arabinose from galactan and pectic galactan. Three enzymes, Arf51B, Arf43C, and Axh43A, liberated arabinose from arabinan. Arf51B and Axh43A in addition released arabinose from soluble wheat arabinoxylan, and Arf43C released arabinose from galactan and pectic galactan. While Arf51B showed the highest specific activity for arabinan, Axh43A displayed the highest activity for arabinoxylan and only a low activity for arabinan. Arf43C showed the highest activity for potato pectic galactan. The mannanase Man26A degraded the β-(1,4)-linked backbones of mannan, galactomannan and glucomannan. In general, the determined activities for the exo-acting enzymes Axh43A, Arf43A, Arf51B, Arf43C, Bxl3B, Bga35A, and Bga2B, were significantly lower than for endo-acting enzymes.

### Biochemical characterisation of hemicellulolytic enzymes

**Fig. 3:**
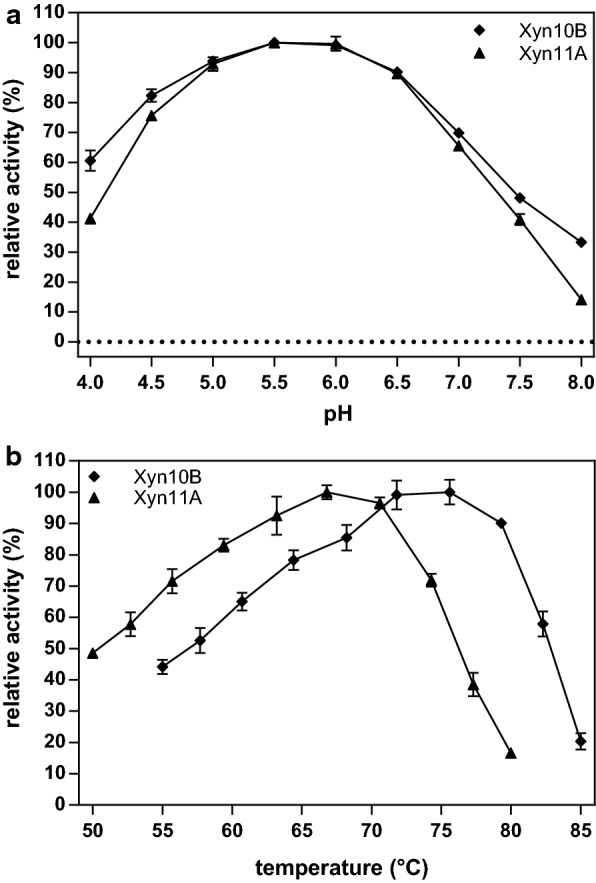
Relative activity of Xyn11A and Xyn10B with 0.5% soluble arabinoxylan at different pH values (**a**) and temperatures (**b**). Experiments were performed for 30 min at **a** 60 °C or **b** at the optimal pH of each enzyme in 0.1 M citrate-phosphate reaction buffer with 0.1 mg/L Xyn10B or 0.05 mg/L Xyn11A

The pH and temperature optima of 13 selected enzymes from *C. stercorarium* with promising hemicellulolytic activities were determined. All enzymes preferred a slightly acidic pH and showed the highest activity between pH 5.0 and pH 6.5. While the pH optima were nearly identical, the temperature optima differed significantly and ranged from 49 to 81 °C (Table 4). Eleven enzymes displayed maximum activity at optimal pH between 57 and 76 °C. The highest temperature optimum of 81 °C was observed for the extracellular Cel9Z, whereas the lowest temperature optimum at 49 °C was found with the intracellular Arf43C. The four xylanases were all most active above 65 °C, with Xyn10B as the enzyme with the highest temperature optimum at 76 °C (Fig. 3). The temperature range in which the activity exceeded 60% of the maximum activity was between 8 and 30 °C, with Bga2B and Arf51B displaying more than 60% activity over a 30 °C wide temperature range.

**Table 4:** Optimal reaction conditions of 13 hemicellulolytic glycoside hydrolases

Enzyme	pH_opt_	pH range	*T*_opt_ (°C)	*T* range (°C)	Substrate
Bga2B	6.0	5.0–6.5	56.8	40–70	Xyloglucan
Bxl3B	5.5	5.0–6.5	65.6	48–72	Glucuronoxylan
Cel9Z	5.5	4.0–6.5	80.6	66–87	β-Glucan (barley)
Xyn10C	6.0	4.5–7.5	70.6	56–77	Arabinoxylan
Xyn10B	5.5	4.0–7.0	75.6	61–79	Arabinoxylan
Xyn10D	6.0	5.0–7.0	65.6	58–66	Arabinoxylan
Xyn11A	5.5	4.5–7.0	66.8	56–74	Arabinoxylan
Man26A	5.5	5.0–6.0	61.8	48–66	Galactomannan
Bga35A	5.5	5.0–6.0	60.6	40–67	Galactan (lupin)
Axh43A	6.0	5.5–6.5	60.6	49–64	Arabinoxylan
Arf43C	6.0	5.5–7.0	49.4	43–64	Pectic galactan (potato)
Abn43A	6.5	6.0–7.0	65.6	51–72	Arabinan
Arf51B	5.0	4.5–5.5	61.8	45–75	Arabinan

The enzymes were incubated with 0.5% of the indicated substrate at pH 4.0 to pH 8.0 (at 60 °C) and between 40 °C and 90 °C (at the enzymes’ optimal pH) for 30 min, 60 min (Bga2B, Bga35A and Arf51B) or 120 min (Bxl3B). Enzyme activities were determined by DNSA-based assay in triplicates. Borders for pH and *T* ranges were set to 60% of the highest activity

### Substrate specificity and activity on oligosaccharides

**Fig. 4:**
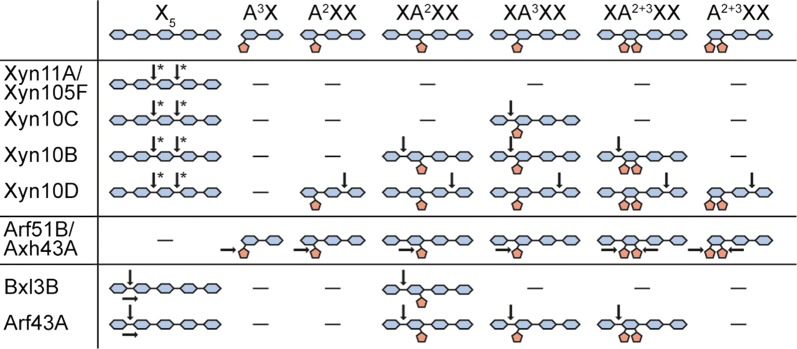
Hydrolysis pattern of investigated arabinoxylanolytic enzymes on purified arabinoxylo- and xylooligosaccharides. Arrows indicate the first cleavage site of the enzymes. Arrows with asterisk indicate potential cleavage sites. Due to the design of the experiment, it was not possible to identify the actual cleavage site. The hydrolysis experiments were performed overnight at 60 °C and pH 6.5 with 2 mg/L enzyme and 20 mg/L pure oligosaccharide or oligosaccharide mixture. Analysis of hydrolysis products was performed using HPAEC-PAD. Xylanases (Xyn), arabinofuranosidases (Arf), arabinoxylan arabinofuranohydrolase (Axh), xylosidases (Xyl)

The secreted proteins of *C. stercorarium* displayed the highest activity for wheat arabinoxylan and several of the recombinant proteins were specific for wheat arabinoxylan. In order to determine the substrate specificity of the xylanases (Xyn11A, Xyn10B–D, Xyn105F), β-xylosidases (Bxl3B, Arf43A) and arabinose-releasing enzymes (Arf51B, Axh43A) in greater depth, purified xylooligosaccharides (XOS) and arabinoxylooligosaccharides (AXOS) were hydrolysed and the degradation products were analysed by HPAEC-PAD (Fig. 4). All five xylanases degraded xylopentaose first to xylotriose and xylobiose and released xylotriose and xylobiose or two xylobiose molecules and xylose as final products. Only the GH10 xylanases Xyn10B, Xyn10C, and Xyn10D were able to partially degrade certain AXOS. Xyn10B and Xyn10C hydrolysed the xylose backbone at the non-reducing end next to an arabinosylated xylose residue, while Xyn10D hydrolysed the reducing end of AXOS, one xylose residue next to the substituted xylose. Xyn10B released the terminal xylose from XA^2^XX, XA^3^XX, and XA^2+3^XX, while Xyn10C was unable to hydrolyse AXOS containing α-(1,2)-linked arabinosylations. Xyn10D cleaved off the terminal xylose at the reducing end of all AXOS with the exception of A^3^X.

Arf51B and Axh43A containing a GH51 and two GH43 modules, respectively, completely cleaved the α-(1,2) and α-(1,3)-linked l-arabinose residue off singly and doubly substituted AXOS releasing xylooligosaccharides and arabinose. Both β-xylosidases, Bxl3B and Arf43A, sequentially degraded xylopentaose to xylose. Arf43A and Bxl3B cleaved off the terminal xylose at the non-reducing end of XA^2^XX, but only Arf43A was able to also hydrolyse XA^3^XX and XA^2+3^XX.

### Product specificity of the xylanases Xyn11A and Xyn10B

**Fig. 5:**
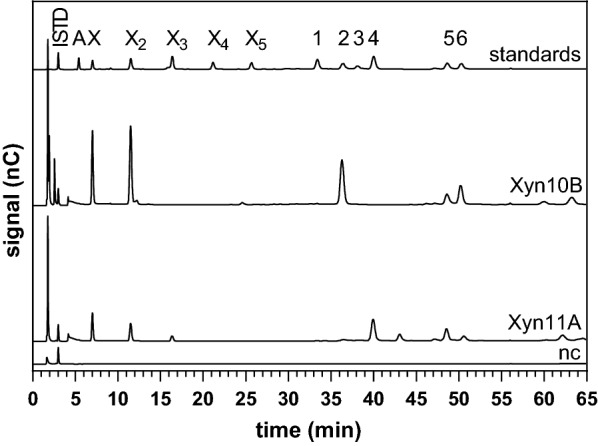
Hydrolysis of 0.5% (w/v) soluble wheat arabinoxylan using Xyn11A and Xyn10B analysed by HPAEC-PAD. The experiments were performed overnight (20 h) at 60 °C with 5 mg/L Xyn10B or Xyn11A. Standards: arabinose (A), xylose (X), xylobiose (X_2_), xylotriose (X_3_), xylotetraose (X_4_), xylopentaose (X_5_), A^2^XX (1), A^3^X (2), XA^2^XX (3), XA^3^XX (4), XA^2+3^XX (5), A^2+3^XX (6), internal standard (ISTD), negative control (nc)

To further investigate the substrate specificities (Figs. 4 and 5) and the product specificities of Xyn11A and Xyn10B, wheat arabinoxylan hydrolysate obtained after enzymatic hydrolysis was analysed by HPAEC-PAD. In agreement with the TLC results, the degradation products of arabinoxylan hydrolysed by Xyn11A and Xyn10B are XOS and AXOS. Xyn11A released xylose, xylobiose and xylotriose, while Xyn10B produced xylose and xylobiose as final products, indicating the degradation of xylotriose. The quantity of released XOS decreased with the product polymerisation degree for Xyn11A, whereas Xyn10B released xylose and xylobiose in similar quantities. Besides the liberation of XOS, Xyn10B hydrolysed wheat arabinoxylan into three known AXOS, A^3^X, XA^2+3^XX, and A^2+3^XX. The amount of released A^3^XX was approximately twice as high as the amount of XA^2+3^XX and A^2+3^XX. Xyn11A produced XA^3^XX, XA^2+3^XX, A^2+3^XX, and an unknown substance with a retention time at 43.1 min. While Xyn10B produced more A^2+3^XX than XA^2+3^XX, Xyn11A produced more XA^2+3^XX. In total, Xyn10B released larger amounts of XOS and AXOS from wheat arabinoxylan, due to its broad substrate specificity, even though the specific activity of Xyn11A was higher.

## Discussion

### Hemicellulose degradation capacity of *C. stercorarium*

Utilisation of renewable biomass for the generation of fuels, electricity, and heat is a climate-friendly addition to the common energy production technologies [59]. In order to permit an energetically and economically efficient process, the degradation of the different polysaccharides in biomass should be as complete as possible. *C. stercorarium*, *Clostridium thermocellum* and closely related species were identified as major hydrolytic bacteria in biogas fermenters [14]. It was shown that a synthetic culture of *C. thermocellum* and *C. stercorarium* degraded 76% of maize silage and performed only slightly less effectively than the best thermophilic bacterial consortium isolated from a biogas plant [14]. Whereas *C. thermocellum* degrades and utilises cellulose with high efficiency, *C. stercorarium* seems to be specialised in the degradation of hemicellulose, since this organism harbours a great number of genes involved in the hydrolysis of hemicellulose in its genome and has the capability to utilise the degradation products, hexoses as well as pentoses [12–14].

Both preparations of extracellular proteins, precipitated from *C. stercorarium* cultures grown on oat spelt xylan or filter paper, degraded 15 of the 26 tested substrates, preferably soluble xylans and barley β-glucan (Fig. 1). The results are in accordance with a previous description of high hydrolytic activities in the culture supernatant of the type strain of *C. stercorarium* for arabinoxylan and β-glucan [12]. The significantly higher activity of the enzyme preparation obtained after growth on oat spelt xylan for mixed linkage glucans as well as the higher activity of the enzymes secreted during cultivation on filter paper for xylans are probably a result of the attachment of the major xylanases and cellulases, respectively, to xylan and cellulose. Xyn11A, Xyn10C, and Cel9Z contain CBMs that bind to the polysaccharides, decreasing the concentrations of these enzymes in the supernatant [60–62]. Although *C. stercorarium* grew well on both carbon sources, it grew faster on oat spelt xylan and secreted 3 times the amount of protein on this substrate, indicating a preference for xylan over filter paper.

Thirty-two of the investigated hemicellulolytic enzymes displayed polysaccharide-degrading activities when produced heterologously while no activity could be determined for the remaining 18 proteins. The reasons could be missing substrates or pH and temperature optima that differ significantly from the applied assay conditions (60 °C, pH 6.0) as shown for RamB, which had a significantly higher activity at 50 °C than at 60 °C. The pH optimum of the previously characterised enzyme Xyn43B is pH 3.5, while the activity of the previously characterised α-d-glucuronidase Agu67A was not detected because the required substrates (for example: 4-*O*-methyl-α-d-xylotriose) were not accessible [28, 30]. Further enzyme activities could have passed undetected due to the limitations of the applied assay conditions or substrates. The recombinant enzymes covered the same activities as the secretome, except for pullulan, pachyman, and polygalacturonic acid and additionally displayed activities for arabinan, mannan, and galactomannan. Five GH13 enzymes, possible pullulanases, were excluded from this investigation [63].

*Clostridium stercorarium* has an effective secretion system for endo-acting glycoside hydrolases, especially for the most important xylanases. However, four of the proteins were apparently not present in the proteome. Whether these proteins are produced in the presence of alternative carbon sources remains to be determined.

### Degradation and debranching of xylans

All xylans share a β-(1,4)-d-xylopyranose backbone, which is substituted by short glycoside and ester groups. Substitutions comprise l-arabinose (Ara), d-glucuronic acid (GlcA) or 4-*O*-methyl-α-d-glucuronic acid (MeGlcA), d-xylose (Xyl), galactose, acetic acid and phenolic acid esters. Xylan in hardwood, usually referred to as 4-*O*-methyl-d-glucurono-d-xylan (MGX), has mainly MeGlcA substitutions attached at position *O*-2 of the xylose chain with an average Xyl:MeGlcA ratio of 10:1. Arabinoxylan (AX), the major hemicellulose component in grasses and cereals, is substituted by α-l-arabinofuranosyl residues at position *O*-2 and/or *O*-3 of the xylose monomers [5].

The apparent importance of xylans as growth substrates for *C. stercorarium* is reflected by its multitude of xylanases. Besides the well-characterised extracellular xylanases Xyn11A, Xyn10B, and Xyn10C, the activity screening revealed three new endo-β-(1,4)-xylanases: Xyn10D, Xyn10E, and Xyn105F. All six xylanases were most active on soluble wheat arabinoxylan. Xyn10B and Xyn10C further showed activity for mixed linkage glucans as previously reported for both enzymes [17]. Xyn11A showed a comparably high activity towards insoluble arabinoxylan due to a high specificity of the three CBM6 modules for insoluble xylan [60]. One possible explanation for the higher activity of Xyn10C for barley β-glucan compared to Xyn10B is that the CBM9 and two CBM22 modules of Xyn10C preferably bind to β-(1,3;1,4)-glucan [61].

The substrate and product specificities of Xyn11A and Xyn10B are in accordance with the product pattern described for GH11 and GH10 xylanases. GH11 xylanases hydrolyse only unsubstituted xylan regions producing longer XOS and AXOS, while GH10 xylanases are able to hydrolyse the xylan backbone at the non-reducing end next to an arabinosylated xylose. GH10 xylanases produce mainly xylose, xylobiose and short AXOS [45, 64]. Unlike Xyn10B, Xyn10C was unable to cleave the terminal xylose off the non-reducing end of AXOS with arabinosylations at position *O*-2. Xyn10D differed significantly and hydrolysed the second β-(1,4)-linkage behind the arabinosylation at the reducing end of AXOS. A similar hydrolytic mechanism was described for the xylanase Xyn10B of *Herbinix hemicellulosilytica* [45]. This highlights the specific role of different xylanases in the degradation of natural hemicelluloses.

To our knowledge, Xyn105F is the first xylanase with a GH105 module. A relatively low but significant activity was measured for soluble arabinoxylan, while Xyn105F was also active on xylopentaose, releasing xylose and xylobiose. Therefore, we discovered a new activity for GH family 105, which until now had exclusively comprised unsaturated rhamnogalacturonyl and β-glucuronyl hydrolases which also might be the main activity of Xyn105F [38, 65]. However, this activity was not determined in this study.

The pH and temperature optima of the four xylanases Xyn11A, Xyn10B, Xyn10C, and Xyn10D are around pH 5.5–6.0 and 65–76 °C, in accordance with previous work [17, 66]. The genes for Xyn11A, Xyn10B, and Xyn10C encode N-terminal signal peptide sequences, and the proteins are extracellular xylanases, abundant in the culture supernatant. In contrast, Xyn10D and Xyn10E seem to be intracellular proteins, while Xyn105F was not produced by *C. stercorarium* under the growth conditions of this study.

Arf51B, Bxl3B, and Arf43A had little activity on xylans and showed α-arabinofuranosidase and β-xylosidase activity towards *p*NP-glycosides, as described previously [17]. Arf43A has previously been characterised as an α-arabinofuranosidase. However, in this work it had higher activity towards *p*NP-β-d-xylopyranoside and, like Bxl3B, released only xylose units from XOS and AXOS [17, 26]. As described previously, Arf51B debranched arabinoxylan but showed a higher activity for arabinan [56]. Arf51B and Axh43A completely de-arabinosylated singly and doubly substituted AXOS, the debranching activity of Axh43A towards soluble arabinoxylan being 130 times higher. Both enzymes belong to the subclass AXHB-md 2,3 of arabinose-releasing enzymes [67]. Axh43A contains a CBM6 module that often binds to β-xylan and presumably enhances the debranching activity towards arabinoxylan [68].

Xylanases, α-arabinofuranosidases, including arabinoxylan arabinofuranohydrolases, and β-xylosidases act synergistically to completely hydrolyse arabinoxylan. Adelsberger et al. [17] showed that Xyn11A or Xyn10C combined with Arf51 and Bxl3B degraded de-esterified arabinoxylan completely to its monosaccharides. We identified at least four new enzymes that are involved in the xylan degradation of *C. stercorarium*. The new enzymes and the detailed information about their substrate and product specificities are helpful for improving the reconstituted enzyme system for xylan hydrolysis. A substitution of Arf51B with Axh43A, for example, could accelerate the debranching of arabinoxylan while Xyn10B and Xyn10D are able to hydrolyse more AXOS than Xyn11A and Xyn10C.

### Degradation of glucan, xyloglucan, and mannan

Mixed linkage glucans are linear polysaccharides comprised of β-(1,4)-linked d-glucose oligomers, which are separated by single β-(1,3)-linkages. Xyloglucans (XG) have a β-(1,4)-d-glucopyranose backbone branched with α-(1,6)-d-xylopyranoside units. The xylopyranoside residues can be substituted by β-(1,2)-linked d-galactopyranose, a further xylopyranose, or other sugars [1, 5]. Galactoglucomannans (GGM) have a linear β-(1,4)-linked backbone comprised of β-d-glucopyranose and β-d-mannopyranose. The main polymeric chain is acetylated and substituted by α-(1,6)-d-galactopyranosyl residues [4, 5]. Galactomannan backbones consist exclusively of β-(1,4)-linked d-mannopyranose units and are also substituted with galactosyl residues. Slightly galactosylated (4–15%) polymers are referred to as glucomannans and mannans [5].

The endo-glucanase Cel9Z was secreted by *C. stercorarium* and showed activity towards mixed linkage glucans and glucomannan. In contrast to previous characterisations, only insignificant activity towards microcrystalline cellulose (Avicel) was determined [18, 20]. The high-temperature optimum of 81 °C can partially be explained by the presence of a thermostabilising CBM3 module, while a second CBM3 module mediates the binding to crystalline cellulose [62]. The determined β-galactosidase and endo-β-mannanase activity of the newly characterised enzymes Bga2B and Man26A, respectively, are in accordance with the common activities for the GH families 2 and 26 [69]. Unlike the other newly characterised β-galactosidases Bga2C–E, Bga2B is able to hydrolyse the β-(1,2)-d-galactopyranosyl substitution of xyloglucan. Man26A contains a CBM35 module that commonly binds to mannan and mannooligosaccharides [70].

### Degradation of arabinan and galactan

Arabinans and (arabino)galactans are side chains of the pectic polysaccharide rhamnogalacturonan I. In addition, α-(1,5)-linked arabinan structures may also exist as free polymers unattached to the pectic domains [71]. Arabinans are composed of an α-(1,5)-l-arabinofuranoside backbone branched with α-(1,2)- and/or α-(1,3)-linked l-arabinosyl oligosaccharides at position *O*-2 or *O*-3. Galactans are linear β-(1,4)-d-galactopyranoside polymers substituted by single α-l-arabinofuranoside units at position *O*-3 [72, 73].

As described above, Arf51B released arabinose from arabinan, probably by cleaving off the α-(1,2)- and α-(1,3)-linked arabinosylations [67]. Four new enzymes for the degradation of arabinan and galactan were characterised: Arf43C, Abn43A, Bga35A, and Gal53A. Arf43C released arabinose from arabinan and galactan, with a higher activity towards galactan, especially pectic galactan. The α-arabinofuranosidase probably prefers or hydrolyses exclusively α-(1,3)-arabinosylations. Abn43A showed endo-α-(1,5)-arabinanase activity in degrading arabinan and also α-(1,3)-arabinofuranosidase activity in debranching galactans. The activities of both enzymes are in accordance with the common activities of GH family 43 [67, 68]. Abn43A and Arf43C or Arf51A combined could be sufficient to hydrolyse arabinan completely to arabinose, as described for combinations of endo-arabinanase and α-arabinofuranosidase [67].

Gal53A showed endo-β-galactanase activity towards galactan and pectic galactan in accordance with the common activity for GH family 53 [38]. The native full-length protein contains a signal peptide, four CBM61 modules, and three S-layer homology modules. The enzyme was secreted by *C. stercorarium* and probably binds to the cell surface [74]. CBM61 binds to β-(1,4)-galactan [75], indicating that the full-length protein has a similar activity to the shortened variant of Gal53A, which was tested here. In accordance with the common activity for GH family 35, Bga35A showed β-galactosidase activity towards galactans [69].

### Hydrolysis of aryl-glycosides

For 15 glycosidases only *p*NP-glycoside-cleaving activities were determined. Beside the previously characterised enzymes Bxl39A, Bgl3Z, Xyl43A, Xyl43B, and Ram78A, the activity screening revealed ten new enzymes: β-d-galactosidases Bga2C, Bga2D, and Bga2E; β-d-xylosidases Bxl43C and Bxl31D; β-d-glucosidase BglA; α-l-arabinofuranosidase ArfD; α-l-rhamnosidase RamB; β-d-glucuronidase Uid2A; and *N*-acetyl-β-d-glucosaminidase Nag3A [16, 17, 23, 28]. Determined activities of Bga2C–E, Uid2A, Bxl43C, and Nag3A are in accordance with the predicted activity of their respective GH families [68, 69, 76]. Interestingly, enzymes of the GH family 31 are often α-glycosidases while Bxl31D hydrolysed exclusively *p*NP-β-d-xylopyranoside, thereby adding a new activity to the GH family 31 [38, 69]. It can be assumed that the aryl-glycosidic activity corresponds to an alkyl-glycosidic activity on polymeric and oligomeric substrates. The enzymes BglA, ArfD, and RamB contain GH modules that have not yet been assigned to a GH family. They could be the first characterised enzymes that define new GH families. To clarify this, further experiments are needed.

## Conclusions

*Clostridium stercorarium* serves as a model organism for the degradation of lignocellulosic biomass, especially for the hydrolysis of hemicellulose. However, only enzymes for the degradation of cellulose and xylan have been well characterised so far [12, 35]. In this study we systematically investigated the majority of glycoside hydrolases from *C. stercorarium* DSM 8532 and we present 20 new enzymes for the bioconversion of hemicellulose. We characterised the first *C. stercorarium* enzymes involved in the degradation of xyloglucan, mannan as well as galacto- and glucomannan, arabinan, and galactan. The determined activities of native extracellular and recombinantly produced enzymes lead to a better understanding of the hemicellulose-degrading capacity of the bacterium and the involved enzymes. Additionally, we discovered new activities not reported before for the GH families 31 and 105 and present three enzymes possibly belonging to new GH families. While the optimal temperature for the majority of the characterised enzymes (57–76 °C) matches the optimum growth temperature of *C. stercorarium* (60–70 °C), the enzymes prefer a slightly more acidic pH than the bacterium, pH 5.0–6.5 instead of pH 7.3 [10].

Enzyme combinations selected from the newly characterised glycoside hydrolases, especially of enzymes with endo- and exo-activity, could lead to a complete degradation of mixed linkage glucan, arabinan or galactan to monosaccharides, as previously described for arabinoxylan [17]. However, further studies will be needed to reconstitute the *C. stercorarium* specific enzyme systems for the complete degradation of defined polysaccharides and hemicellulose. The degradation of xyloglucan or mannan requires at least one xyloglucanase and one β-mannosidase, respectively, activities that have not yet been identified in *C. stercorarium* [4]. In combination with activity assays, proteomic analyses of the *C. stercorarium* secretomes secreted in the presence of various polysaccharides could be applied to identify the enzymes the bacterium employs to their degradation. The hydrolysis of complex polysaccharides requires further accessory enzymes like carbohydrate esterases (CE) and polysaccharide lyases (PL), which were not subject of this study [8].

Detailed substrate and product specificities as determined for xylanases, α-arabinofuranosidases and β-xylosidases increase the understanding of the reaction mechanism and provide a promising platform for biotechnological applications. Such applications often require highly specific activities. Especially α-l-arabinofuranosidases with the ability to hydrolyse single and double substitutions, like Arf51B and Axh43A, are of great interest because these substitutions inhibit or even prevent arabinoxylan and pectin from complete degradation to their monomers [2, 67].

Bacteria active in hemicellulose degradation such as *C. stercorarium* are a rich source of specific enzyme activities needed for industrial applications. By cloning hitherto unknown gene families, as done in this study, even new protein families can be detected.

## Additional files

**Additional file 1.** This file contains additional tables and figures as follows: (i) **Table S1.** PCR primer for the amplification of the 50 selected glycoside hydrolase genes of *C.* *stercorarium*. (ii) **Table S2.** Polysaccharide and *p*-nitrophenyl substrates including the final concentration in enzymatic assays. (iii) **Table S3.** Glycoside hydrolase (GH) families present in the *C.* *stercorarium* genome. (iv) **Figure S1.** SDS-PAGEs of 10 examples of the 50 *C.* *stercorarium* proteins recombinantly produced by *E.* *coli* and purified by IMAC. (v) **Figure S2.** Schematic structure of the glycoside hydrolases with proven activity from *C.* *stercorarium*. (vi) **Table S4.** Studied enzymes in the secretome or intracellular proteome of *C.* *stercorarium* analysed by LC-MS/MS. (vii) **Figure S3.** Hydrolytic products of different polysaccharides analysed by Thin-layer chromatography (TLC). Polysaccharides were hydrolysed by a) Xyn10C, b) Xyn11A, c) Xyn10D, d) Arf51B, e) Arf43C, f) Axh43A, g) Abn43A, h) Cel9Z, i) Man26A, j) Bga35A, k) Bga2B. (viii) **Figure S4.** Relative activity of characterised enzymes at different pH. pH profiles of a) xylanases Xyn11A & Xyn10B-D, b) Axh43A and α-arabinofuranosidases Arf51B & Arf43C, c) β-galactosidases Bga2B & Bga35A, d) Bxl3B, Cel9Z, Man26A, and Abn43A. (ix) **Figure S5.** Relative activity of characterised enzymes at different temperatures. Temperature profiles of a) xylanases Xyn11A & Xyn10B-D, b) Axh43A and α-arabinofuranosidases Arf51B & Arf43C, c) β-galactosidases Bga2B & Bga35A, d) Bxl3B, Cel9Z, Man26A, and Abn43A.

**Additional file 2.** This file contains the complete processed data set of the LC-MS/MS proteome analysis: LC–MS/MS data of the secretome (SU) and the intracellular proteome (PE) of *C. stercorarium*.

## Abbreviations

Abn: arabinanase
Arf: α-arabinofuranosidase
Axh: arabinoxylan arabinofuranohydrolase
Bga: β-galactosidase
Bgl: β-glucosidase
Bxl: β-xylosidase
CAZy: Carbohydrate-Active enZYme database
CBM: carbohydrate binding module
Cel: cellulase
DNSA: 3,5-dinitrosalicylic acid
Gal: galactanase
GH: glycoside hydrolase
HPAEC-PAD: high-performance anion-exchange chromatography with pulsed amperometric detection
iBAQ: intensity based absolute quantification
ISTD: internal standard
Man: mannanase
MOPS: 3-(*N*-morpholino)propanesulfonic acid
Nag: *N*-acetyl-β-d-glucosaminidase
Ram: rhamnosidase
TLC: thin-layer chromatography
Uid: β-d-glucuronidase
Xyl: xylosidase
Xyn: xylanase
