# Supplementary material for: The hemicellulose-degrading enzyme system of the thermophilic bacterium Clostridium stercorarium: comparative characterisation and addition of new hemicellulolytic glycoside hydrolases
Source: Biotechnol Biofuels. 2018 Aug 23;11:229. doi: 10.1186/s13068-018-1228-3 (PMC6106730; doi:10.1186/s13068-018-1228-3)
Supplement: Supplementary file 1 — Additional file 1. This file contains additional tables and figures as follows: (i) Table S1. PCR primer for the amplification of the 50 selected glycoside hydrolase genes of C. stercorarium. (ii) Table S2. Polysaccharide and p-nitrophenyl substrates including the final concentration in enzymatic assays. (iii) Table S3. Glycoside hydrolase (GH) families present in the C. stercorarium genome. (iv) Figure S1. SDS-PAGEs of 10 examples of the 50 C. stercorarium proteins recombinantly produced by E. coli and purified by IMAC. (v) Figure S2. Schematic structure of the glycoside hydrolases with proven activity from C. stercorarium. (vi) Table S4. Studied enzymes in the secretome or intracellular proteome of C. stercorarium analysed by LC-MS/MS. (vii) Figure S3. Hydrolytic products of different polysaccharides analysed by Thin-layer chromatography (TLC). Polysaccharides were hydrolysed by a) Xyn10C, b) Xyn11A, c) Xyn10D, d) Arf51B, e) Arf43C, f) Axh43A, g) Abn43A, h) Cel9Z, i) Man26A, j) Bga35A, k) Bga2B. (viii) Figure S4. Relative activity of characterised enzymes at different pH. pH profiles of a) xylanases Xyn11A & Xyn10B-D, b) Axh43A and α-arabinofuranosidases Arf51B & Arf43C, c) β-galactosidases Bga2B & Bga35A, d) Bxl3B, Cel9Z, Man26A, and Abn43A. (ix) Figure S5. Relative activity of characterised enzymes at different temperatures. Temperature profiles of a) xylanases Xyn11A & Xyn10B-D, b) Axh43A and α-arabinofuranosidases Arf51B & Arf43C, c) β-galactosidases Bga2B & Bga35A, d) Bxl3B, Cel9Z, Man26A, and Abn43A. [file 13068_2018_1228_MOESM1_ESM.pdf]

The hemicellulose-degrading enzyme system of the thermophilic bacterium *Clostridium stercorarium* – comparative characterisation and addition of new hemicellulolytic glycoside hydrolases

- Additional file 1 -

Broeker[1], Jannis (jannis.broeker@tum.de), Mechelke[1], Matthias (matthias.mechelke@tum.de), Baudrexl[1], Melanie (melanie.baudrexl@tum.de), Mennerich, Denise (denise.mennerich@tum.de), Hornburg[2,3], Daniel (hornburg@stanford.edu), Mann[3], Matthias (mmann@biochem.mpg.de), Schwarz[1], Wolfgang H. (wolfgang.schwarz@tum.de), Liebl[1], Wolfgang (wliebl@wzw.tum.de), Zverlov[1,4*], Vladimir V. (vladimir.zverlov@tum.de)

[1] Department of Microbiology, TUM School of Life Sciences Weihenstephan, Technical University of Munich, Emil-Ramann-Str. 4, 85354 Freising, Germany

[2] Present address: School of Medicine, Stanford University, 94305 Stanford, CA, USA

[3] Max Planck Institute of Biochemistry, Am Klopferspitz 18, 82152 Martinsried, Germany

[4] Institute of Molecular Genetics, Russian Academy of Science, Kurchatov Sq. 2, 123182 Moscow, Russia

[*] Corresponding author: Dr. Vladimir V. Zverlov

email: vladimir.zverlov@tum.de, Tel.: +49-8161-715474

Table S1 PCR primer for the amplification of the 50 selected glycoside hydrolase genes of *C. stercorarium*.

accession number	protein name	sense primer (5'-3')	antisense primer (5'-3')
AGC67981.1	Bga2B	… AACAGGAAAGTGTTGTTCAA	… TTTCTCAAACTTAAACCATCCG
AGC67186.1	Bga2E	… GACGTAAATACCATATCC	… GCCAAGCACTGTTTCAGGAT
AGC67268.1	Bga2D	… AATAGAGAAATTTTGCATC	… AAAACCGTTTTTTACAGGTATTA
AGC67637.1	Bga2C	… CTGCCCGTAAAAAAATACTG	… TTCTATCTGTTCACGAATAATG
AGC68382.1	Uid2A	… GATACGAAGCATGTGG	… ATCTTTTATAGAAGCATAAAATTC
AGC67275.1	Bxl3B	… GAAAACAAACCTGTTTATCT	… CTGCACGCGCAGCACGG
AGC67337.1	Bgl3Z	… GTAATACCAATTGTTGCAAG	… TTTAATATTTGCATTCAGTGTATC
AGC67350.1	-	… ACTTTGAAGGAAAAGATAG	… ATACATGCCATCATTTTTCAGTA
AGC68204.1	-	… ACGGTAAATAACAAAAGCCG	… TGTAAATTTAAAATTACTGATATTGGC
AGC68338.1	Nag3A	… GAACATGAAAATTTTCCGACTG	… GTTACCGAAATTTCTCACTCTT
AGC68873.1	Cel9Z	… GCAGGATATAATTATGGGG	… CGGTTCAATTCCACTGACCA
AGC67515.1	Xyn10E	… TTGACGGTAAAGGTTAAAAT	… TTTACCCATGATTACTTTCTTC
AGC67677.1	Xyn10C	… GAAACAACGGTTTATCATGAG	… TCTTAATCTTAAAAATGCCTC
AGC67715.1	Xyn10B	… TTTAACGATCAAACTTCTGCTG	… TTCCCGCAACCGTGAAGGA
AGC67759.1	Xyn10D	… GAAAATAAAACAGGCGGC	… ACTGTTTAAAATTATTTTTTCCTCA
AGC68909.1	Xyn11A	… GGGCGAATAATTTACGACAATG	… AGTTCCTGATTTTGAGAATACAA
AGC68130.1	Man26A	… ACCGGGAGGCAAAAAGGTTT	… TTTTCCGCGATATTTTTTCAAATC
AGC68671.1	-	… GACAAAAACAAGGTTGC	… GTCGATTTTTGTAAGCTTATAAA
AGC67830.1	-	… GGAAAAGTAAATATCGAAC	… TATGCCTTCCATTTTTTCTACC
AGC67947.1	-	… AGCTATACTACGGTTTT	… CTCAGTGCAGTCGGTTAATC
AGC67128.1	-	… GACCGGAAAAAATATCTTG	... GTCCACCTGAATCTTAAACAC
AGC68208.1	Bxl31D	… AAATTCAGTGATGGTTACTG	… GAACCGTTCATTAACCGTTAT
AGC69232.1	Bga35A	… GCGGTTTATCATTTGGA	… CATTATTTTAAAAACTGCTTCATG
AGC68033.1	-	… CCGTTCAGTAAGGAATGGG	… GGATTTTATCCTGAGCCTTAA
AGC67890.1	Bxl39A	… GCAAGGGAAATCACAATA	… TTCAAGTCCAAAAAATTCATCC
AGC67716.1	Axh43A	… AATAAATACCGGGAAACATCC	… TTCGCTGAAATACCAGTAATC
AGC67945.1	Arf43A	… TTGAAAAATAACTCTTCAGAACC	… TCCTCTGCCGGTACTACC
AGC68110.1	Arf43C	… CAGAATTCGAACATAAGAAC	… TTCATAAACCTGTTTTTGATACTC
AGC68111.1	Abn43A	… GAAACGAAGGATAATCGTGC	… TTCAATGGCCCAAATACCGC
AGC67521.1	Xyl43B	… CAGCCTGACAACAAAC	… TTCAAATTCATATCTGAACCAG
AGC67885.1	Xyl43A	… AGAAAACAGAGATTCAACC	… GTCAGTACACAGAGTGAATG
AGC69509.1	Bxl43C	… CGTTACCATAATCCTAT	… CAGACTTATATCTTCTCTGTC
AGC67626.1	Arf51B	… GCCAAATACGTAATCAAC	… GTCAACCGTAATGCTGACTAC
AGC68692.1	Gal53A	… TACTGCGAAGGAAAGGAGG	… TTCAGTTTCAGGTTCAGGTTC
AGC69355.1	Agu67A	… AAACTGCCAAATTTCGGA	… ATAAATTGTTCTCCCTTTTTCAT
AGC68061.1	Ram78A	… AATCCTATGGGGTTTGTCATT	… TTCCACAGTAACTTTGCTGAG
AGC69452.1	-	… ATTGAAATTTCCGCACAG	… CCACAGAAGTATTCCCAATTC
AGC67127.1	-	… GCGGACGCTGAAGGG	… CGCATTAAAACCGGGATTCAT
AGC68039.1	Xyn105F	… GAAAATTATATTGATCTTGC	… TCCCCCGCTGCCTAAAGC
AGC67892.1	-	… CAGGAAAAAAAAGAAACC	… TTTCTTTGAAACGCTTTTCAGC
AGC67946.1	-	… AAGAAATACGACAATAATTA	… TTTTTCCATTTCAGAGCATGC
AGC68044.1	-	… GGTTATTATTTTCCGGATG	… AATTCCTCCGTTATCAAGCC
AGC68046.1	-	… TCTGTAAACGTGGAAATG	… AATGAGCTCGGTCAGCATAA
AGC67967.1	-	… GCTGAGATCCTGTTCAGGG	… TCCGAGCAGCTTTTCCTTCA
AGC67053.1	-	… GTGAAAATTATGGAGCATGTAT	… TCTGGCTCGTATCCATACCA
AGC67292.1	-	… TTAACCAACACGGAAAAAT	… GCAGAAAGCAACAGGCAAC
AGC67072.1	RamB	… TTTTTTAAAAATAGCCGGG	… TTCAATATATATAAGCGGGGC
AGC68062.1	BglA	… AATTCTGTGAGCGTGG	… ATATTGGCTACAGGTAATCAAA
AGC69032.1	-	… GAATCCACGAAGTTGG	… AACGGGATATATTTTAACAGGC
AGC69275.1	ArfD	… AGGAACTGGTCATTTACAG	… GTTTGTTTCATATACCTTGACC

Ligation in the pET-24c(+) vector war performed with Gibson Assembly. Therefore sense and antisense primer have a 5'-overlap with the vector: sense primer: TTAAGAAGGAGATATACATATG… (22 bp), antisense primer: GTGGTGGTGGTGGTGCTCGA… (20 bp).

Table S2 Polysaccharide and *p*-nitrophenyl substrates including the final concentration in enzymatic assays.

**polysaccharides**	**final conc.**	***p*NP-glycosides**	**final conc.**
arabinan	0.5% w/v	α-L-arabinofuranoside	4 mM
arabinoxylan (wheat, soluble)	0.5% w/v	α-D-glucopyranoside	4 mM
arabinoxylan (wheat, insoluble)	0.5% w/v	β-D-glucopyranoside	4 mM
4-O-methyl-glucuronoxylan	0.5% w/v	α-D-galactopyranoside	4 mM
xylan (birch)	0.5% w/v	β-D-galactopyranoside	4 mM
xylan (oat spelt)	0.5% w/v	α-D-mannopyranoside	4 mM
xyloglucan	0.5% w/v	β-D-mannopyranoside	4 mM
avicel	0.25% w/v	α-L-rhamnopyranoside	4 mM
β-glucan (barley)	0.5% w/v	α-D-xylopyranoside	4 mM
curdlan	0.5% w/v	β-D-xylopyranoside	4 mM
laminarin	0.5% w/v	β-D-glucuronide	4 mM
lichenin	0.5% w/v	N-acetyl-β-D-glucosaminide	4 mM
pachyman	0.5% w/v		
pullulan	0.5% w/v		
arabinogalactan (larch)	0.5% w/v		
galactan (potato)	0.5% w/v		
galactan (lupin)	0.5% w/v		
pectic galactan (potato)	0.5% w/v		
pectic galactan (lupin)	0.5% w/v		
polygalacturonic acid	0.5% w/v		
mannan	0.5% w/v		
mannan (ivory nut)	0.5% w/v		
galactomannan (guar)	0.5% w/v		
glucomannan (konjac)	0.125% w/v		
gum arabic (acacia)	0.5% w/v		
inulin (dahlia tubers)	0.5% w/v		
sinistrin	0.5% w/v		
rhamnogalacturonan I	0.5% w/v		
chitosan	0.5% w/v		

The polysaccharides used in this study were purchased from: Megazyme (Bray, Ireland): arabinan, wheat arabinoxylan (soluble and insoluble), xyloglucan, barley β-glucan, pachyman, arabinogalactan, potato and lupin galactan and pectic galactan, mannan, ivory nut mannan, galactomannan, glucomannan and rhamno-galacturonan I; Sigma-Aldrich (St. Louis, USA): birch xylan, oat spelt xylan, lichenin, polygalacturonic acid, gum arabic, inulin and chitosan; Serva electrophoresis (Heidelberg, Germany): curdlan and avicel; ICN Biochemical (Irvine, USA): pullulan; Alfa Aesar (Ward Hill, USA): laminarin; and Fresenius Kabi (Graz, Austria): sinistrin. Glucuronoxylan were bought from Sigma-Aldrich and Carbosynth (Compton, GB). The *p*NP-glycosides were purchased from: Carbosynth: α-L-arabinofuranoside, β-D-galactopyranoside, α-D-mannopyranoside, β-D-manno-pyranoside and α-D-xylopyranoside; Sigma-Aldrich: α-D-glucopyranoside, α-L-rhamnopyranoside, β-D-xylo-pyranoside and β-D-glucuronide; Alfa Aesar: β-D-glucopyranoside; Serva electrophoresis: α-D-galactopyranoside; and AppliChem (Darmstadt, Germany): N-acetyl-β-D-glucosaminide.

Table S3 Glycoside hydrolase (GH) families present in the *C. stercorarium* genome.

selected GH families:	GH2 (5), GH3 (5), GH9, GH10 (4), GH11, GH26, GH27, GH28 (2), GH29, GH31, GH35, GH38, GH39, GH43 (7), GH51, GH53, GH67, GH78, GH88, GH95, GH105 (5), GH115, GH127 (2), GHnc (4)
excluded GH families:	GH4, GH13 (5), GH15, GH18 (3), GH23 (2), GH36 (2), GH48, GH94 (2), GH112, GH130

Enzymes within 24 of 34 GH families were selected and produced in *E. coli*. The number of enzymes is indicated in brackets behind the GH family, if more than one enzyme is present.

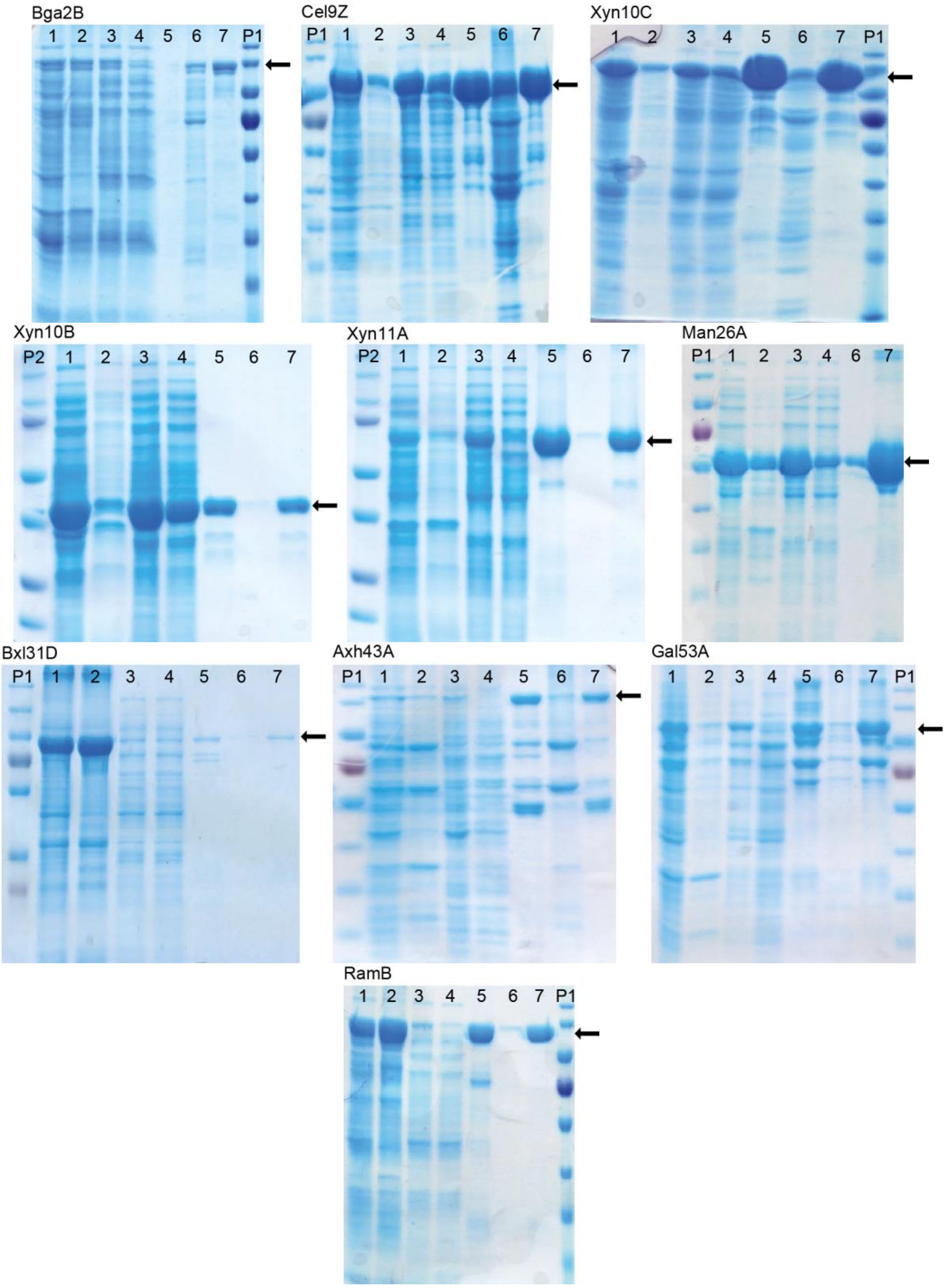

Figure S1 SDS-PAGEs of 10 examples of the 50 *C. stercorarium* proteins recombinantly produced by *E. coli* and purified by IMAC. Purification steps comprises: crude cell extract (1), resuspended cell extract pellet (2), supernatant of the cell extract after centrifugation (3), flow through of IMAC (4) eluate of the IMAC (5), resuspended precipitate of the heat denaturation (6), purified protein solution (7), protein standards with 180, 130, 100, **70**, 55, 40, 35 and 25 kDa (P1) or 250, 150, 100, **75**, 50, 37, **25** and 20 kDa.

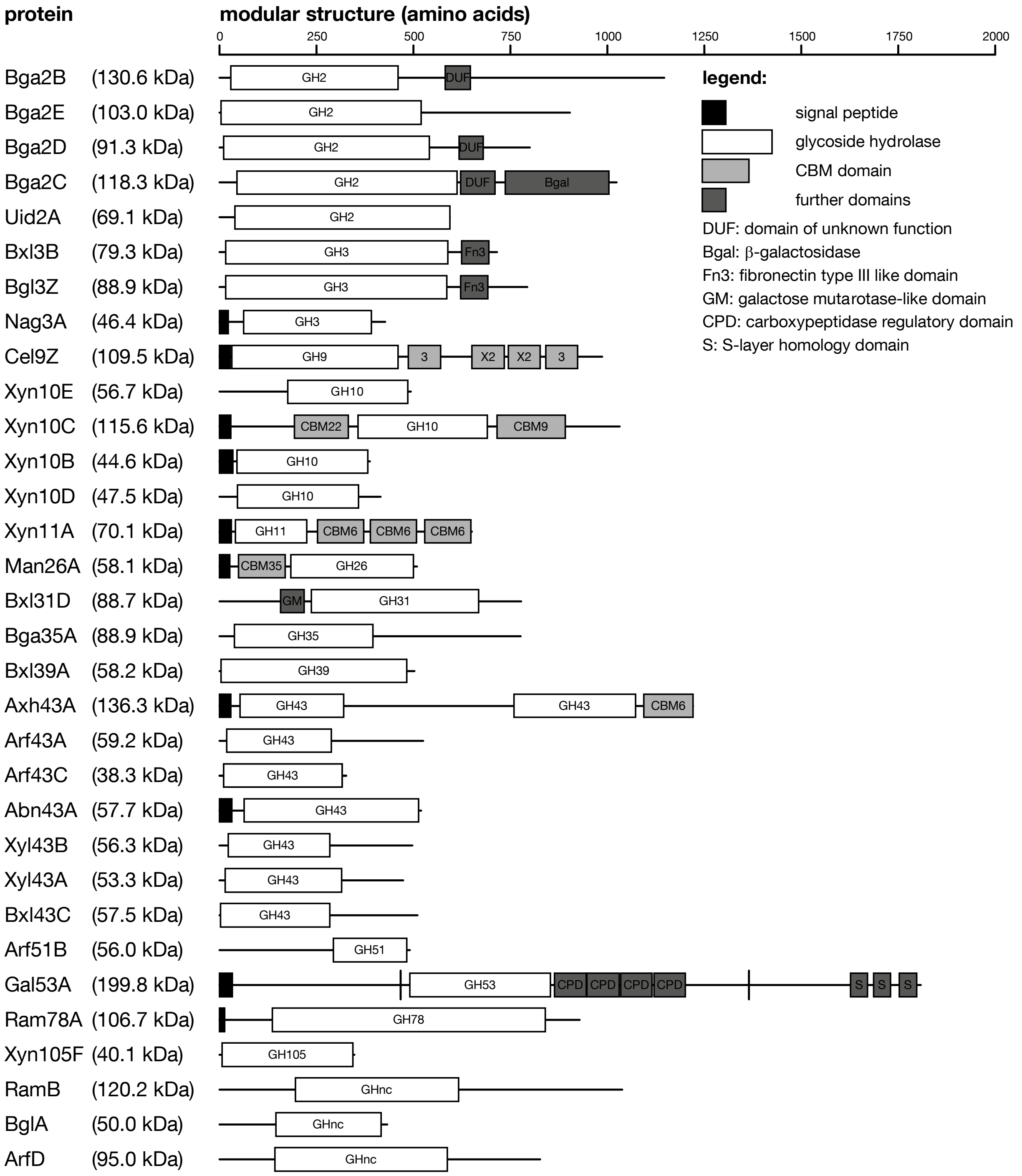

Figure S2 Schematic structure of the glycoside hydrolases with proven activity from *C. stercorarium*. Molecular masses are given in brackets. The size of each enzyme and domain is scaled according to the corresponding amino acid chain length. Classification of GH and CBM modules were obtained from the CAZy database [40]. Domain structure was obtained from the Pfam database [58]. The CAZy database lists only the two CBM3 modules for Cel9Z whereas the four listed CBM61 modules of Gal53A and the second CBM22 module of Xyn10C could not be identified using the Pfam database. Protein Gal53A was shortened in PCR at indicated (I) sites.

Table S4 Studied enzymes in the secretome or intracellular proteome of *C. stercorarium* analysed by LC-MS/MS.

protein name	iBAQ SU	rank SU	iBAQ PE	rank PE
Bga2B	9,47	32 %	12,32	21 %
Bga2E	6,35	67 %	7,47	80 %
Bga2D	3,22	93 %	6,37	88 %
Bag2C	6,66	63 %	11,37	30 %
Uid2A	8,26	44 %	11,85	26 %
Bxl3B	7,12	57 %	8,48	69 %
Bgl3Z	11,16	16 %	12,67	18 %
AGC67350.1	3,44	92 %	8,97	63 %
AGC68204.1	6,40	66 %	9,54	56 %
Nag3A	4,80	81 %	7,88	75 %
Cel9Z	16,11	1 %	4,95	96 %
Xyn10E	5,48	75 %	8,98	63 %
Xyn10C	11,38	14 %	9,20	60 %
Xyn10B	9,07	35 %	-	-
Xyn10D	10,63	19 %	13,26	13 %
Xyn11A	14,27	3 %	-	-
Man26A	6,51	65 %	5,31	95 %
AGC68671.1	8,18	45 %	10,19	46 %
AGC67830.1	9,88	28 %	11,90	25 %
AGC67947.1	11,05	17 %	12,61	18 %
AGC67128.1	6,57	64 %	8,43	70 %
Bxl31D	-	-	5,92	91 %
Bga35A	6,28	68 %	8,86	64 %
AGC68033.1	6,11	69 %	7,66	78 %
Bxl39A	8,77	38 %	12,14	23 %
Axh43A	-	-	-	-
Arf43A	10,04	26 %	11,40	30 %
Arf43C	-	-	4,39	97 %
Abn43A	5,17	77 %	6,98	84 %
Xyl43B	6,57	64 %	10,25	46 %
Xyl43A	9,34	32 %	11,84	26 %
Bxl43C	5,71	73 %	9,49	57 %
Arf51B	10,46	21 %	12,99	15 %
Gal53A	7,64	52 %	5,46	93 %
AGC69355.1	6,63	63 %	10,79	38 %
Ram78A	2,46	96 %	7,70	77 %
AGC69452.1	8,94	36 %	11,15	33 %
AGC67127.1	-	-	3,42	99 %
Xyn105F	-	-	-	-
AGC67892.1	-	-	-	-
AGC67946.1	9,49	31 %	12,18	22 %
AGC68044.1	5,05	78 %	9,52	56 %
AGC68046.1	-	-	9,49	57 %
AGC67967.1	7,90	50 %	10,48	42 %
AGC67053.1	7,63	52 %	10,54	41 %
AGC67292.1	0,86	100 %	3,55	99 %
RamB	1,80	98 %	6,09	90 %
BglA	-	-	-	-
AGC69032.1	4,62	83 %	8,64	67 %
ArfD	4,30	86 %	7,95	74 %

C. stercorarium was grown in GS2 medium with 0.5% w/v cellobiose for 24 h. The protein length normalised log2 protein intensities (iBAQ) and the percentage rank of the protein in the secretome (SU) and intracellular proteome (PE) are given. Some proteins weren’t detected (-).

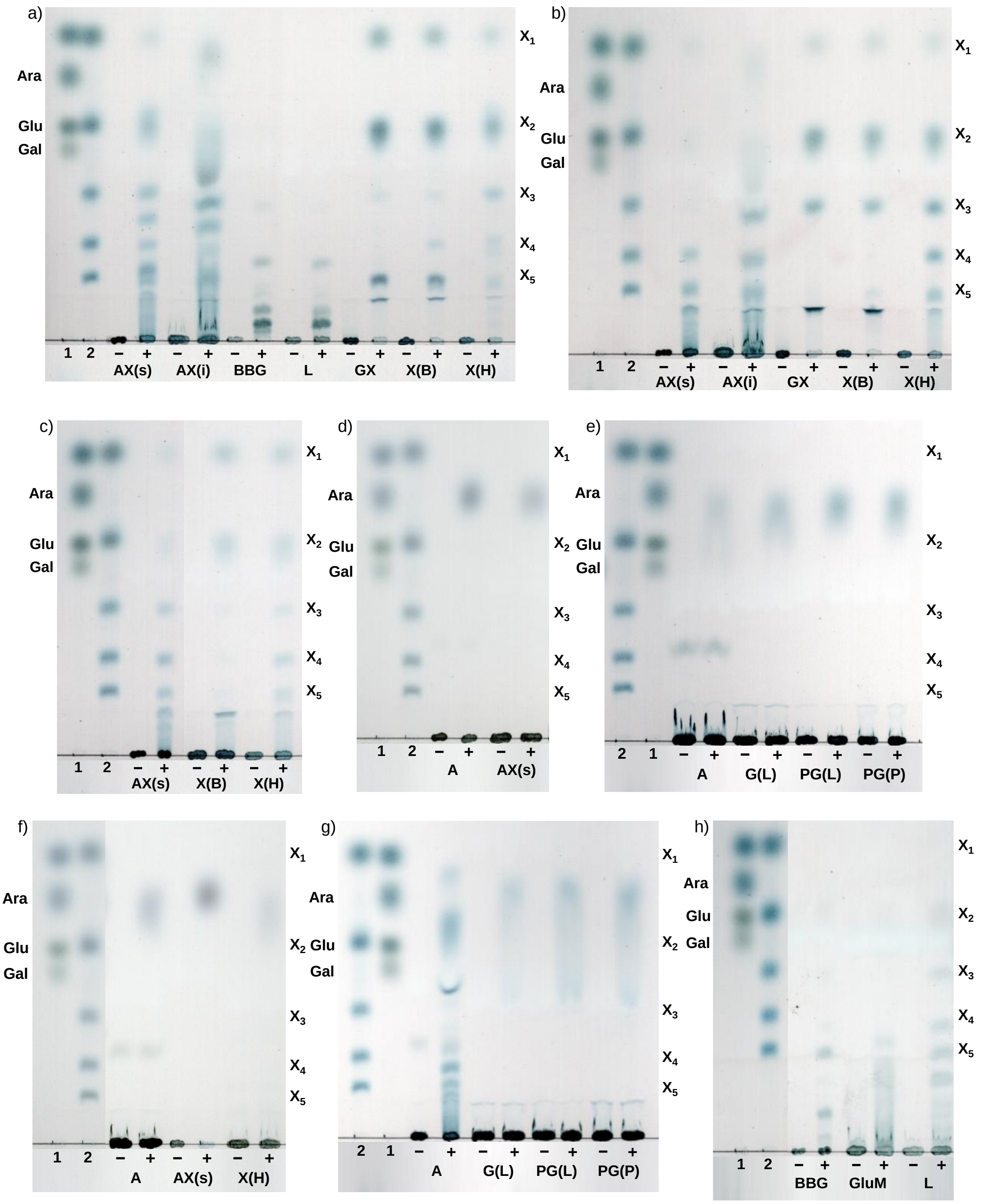
a)
Ara
Glu
Gal
X_1
X_2
X_3
X_4
X_5
1 2 − + − + − + − + − + − + − +
AX(s) AX(i) BBG L GX X(B) X(H)
b)
Ara
Glu
Gal
X_1
X_2
X_3
X_4
X_5
1 2 − + − + − + − + − +
AX(s) AX(i) GX X(B) X(H)
c)
Ara
Glu
Gal
X_1
X_2
X_3
X_4
X_5
1 2 − + − + − +
AX(s) X(B) X(H)
d)
Ara
Glu
Gal
X_1
X_2
X_3
X_4
X_5
1 2 − + − +
A AX(s)
e)
Ara
Glu
Gal
X_1
X_2
X_3
X_4
X_5
2 1 − + − + − + − +
A G(L) PG(L) PG(P)
f)
Ara
Glu
Gal
X_1
X_2
X_3
X_4
X_5
1 2 − + − + − +
A AX(s) X(H)
g)
Ara
Glu
Gal
X_1
X_2
X_3
X_4
X_5
2 1 − + − + − + − +
A G(L) PG(L) PG(P)
h)
Ara
Glu
Gal
X_1
X_2
X_3
X_4
X_5
1 2 − + − + − +
BBG GluM L

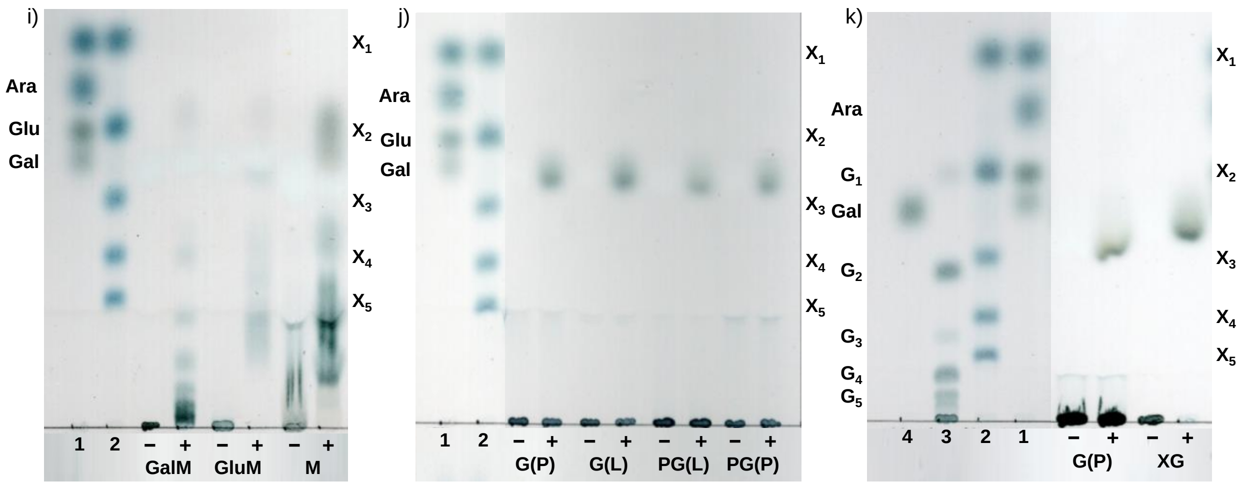

Figure S3 Hydrolytic products of different polysaccharides analysed by Thin-layer chromatography (TLC). Polysaccharides were hydrolysed by a) Xyn10C, b) Xyn11A, c) Xyn10D, d) Arf51B, e) Arf43C, f) Axh43A, g) Abn43A, h) Cel9Z, i) Man26A, j) Bga35A, k) Bga2B. The experiments were performed overnight (16 h) at pH 6.5 and 60 °C with 5.0 mg/L enzyme and 0.5% substrate in 0.1 M MOPS reaction buffer. Arabinan (A), soluble wheat arabinoxylan (AX(s)), insoluble wheat arabinoxylan (AX(i)), barley β-glucan (BBG), galactan lupin (G(L)), galactan potato (G(P)), galactomannan (GalM), glucomannan (GluM), glucuronoxylan (GX), lichenin (L), mannan (M), pectic galactan lupin (PG(L)), pectic galactan potato (PG(P)) xylan (birch) (X(B)), xylan (oat spelt) (X(H)), xyloglucan (XG) negative control (-), hydrolysate (+), standards: xylose, arabinose, glucose, galactose (1), xylose, xylobiose, xylotriose, xylotetraose, xylopentatose (2), glucose, cellobiose, cellotriose, cellotetraose, cellopentose (3), galactose (4).

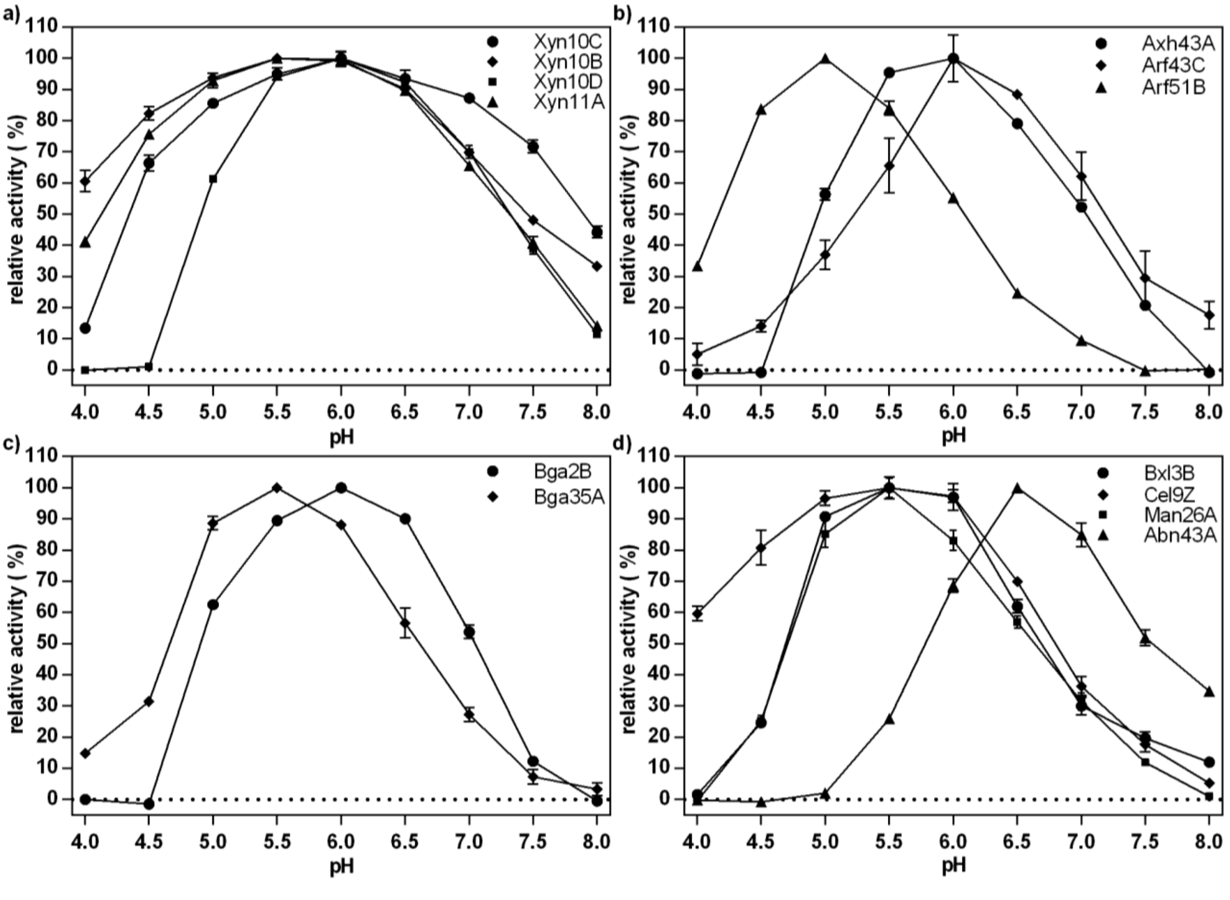

Figure S4 Relative activity of characterised enzymes at different pH. pH profiles of a) xylanases Xyn11A & Xyn10B-D, b) Axh43A and α-arabinofuranosidases Arf51B & Arf43C, c) β-galactosidases Bga3B & Bga35A, d) Bxl3B, Cel9Z, Man26A, and Abn43A. Experiments were performed for 30 min, 60 min (Bga2B, Bga35A and Arf51B) or 120 min (Bxl3B) at 60 °C in 0.1 M citrate-phosphate reaction buffer with 0.05 mg/L Xyn11A, 0.1 mg/L Xyn10B, 0.5 mg/L Xyn10C & Cel9Z, 0.6 mg/L Man26A, 1.0 mg/L Abn43A, 1.5 mg/L Axh43A, 5.0 mg/L Xyn10D & Arf43C, 8.0 mg/L Bga2B, 10.0 mg/L Arf51B & Bga35A and 24.0 mg/L Bxl3B.

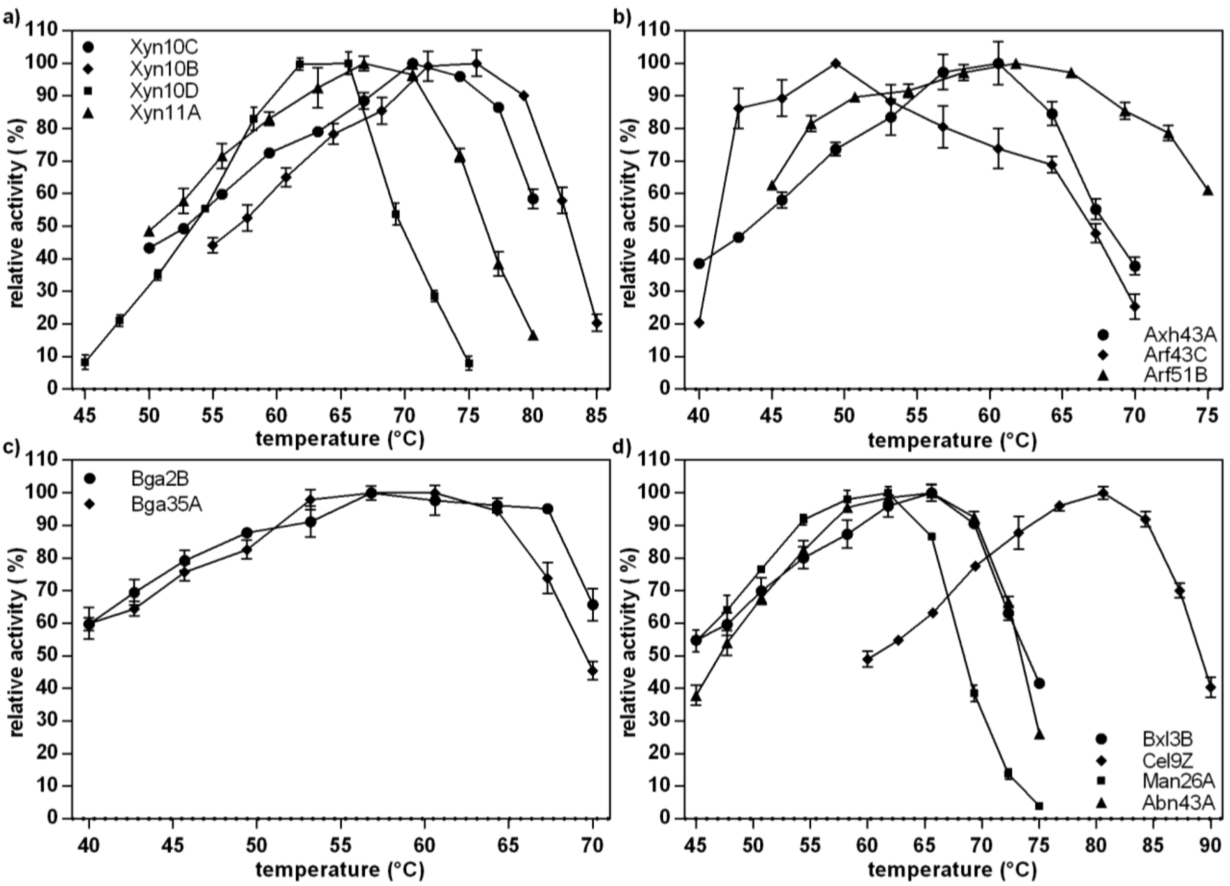

Figure S5 Relative activity of characterised enzymes at different temperatures. Temperature profiles of a) xylanases Xyn11A & Xyn10B-D, b) Axh43A and α-arabinofuranosidases Arf51B & Arf43C, c) β-galactosidases Bga2B & Bga35A, d) Bxl3B, Cel9Z, Man26A, and Abn43A. Experiments were performed for 30 min, 60 min (Bga2B, Bga35A and Arf51B) or 120 min (Bxl3B) at the optimal pH of each enzyme in in 0.1 M citrate-phosphate reaction buffer with 0.05 mg/L Xyn11A, 0.1 mg/L Xyn10B, 0.5 mg/L Xyn10C & Cel9Z, 0.6 mg/L Man26A, 1.0 mg/L Abn43A, 1.5 mg/L Axh43A, 5.0 mg/L Xyn10D & Arf43C, 8.0 mg/L Bga2B, 10.0 mg/L Arf51B & Bga35A and 24.0 mg/L Bxl3B.

References:

38. Architecture et Fonction des Macromolécules Biologiques: Carbohydrate-Active Enzymes database. http://www.cazy.org/ (2014). Accessed 24 Jul 2014.

58. European Bioinformatics Institute: Pfam database. https://pfam.xfam.org/ (2017). Accessed 24 Jul 2017.
